# 3D electron diffraction shows structural flexibility in rare Zn(ii) paddlewheel-based metal–organic polyhedra

**DOI:** 10.1039/d6cc04492a

**Published:** 2026-08-12

**Authors:** Matthew P. Snelgrove, Beatriz Doñagueda Suso, Calum S. Sangster, Megan G. Wilkinson, Emma Regincos Marti, Jeremiah P. Tidey, Alan R. Kennedy, Simon Parsons, Ashleigh J. Fletcher, Gavin A. Craig

**Affiliations:** a Department of Pure and Applied Chemistry, University of Strathclyde Glasgow G1 1RX UK gavin.craig@strath.ac.uk; b EaStCHEM, School of Chemistry and Centre for Science at Extreme Conditions, University of Edinburgh Edinburgh EH9 3FJ UK; c Department of Physics, University of Warwick Gibbet Hill Road Coventry CV4 7AL UK; d Department of Chemical and Process Engineering, University of Strathclyde Glasgow G1 1XJ UK

## Abstract

Zinc paddlewheels are widely found in metal–organic frameworks, but found more sparingly in porous molecules. Here we report some of the first examples of metal–organic polyhedra containing zinc paddlewheels, where structural adaptations in the cages have been observable thanks to the use of 3D electron diffraction.

## Abstract 2

3D ED is used to study the structures of solvent-exchanged phases in zinc paddlewheel-based MOPs.

## Graphical abstract

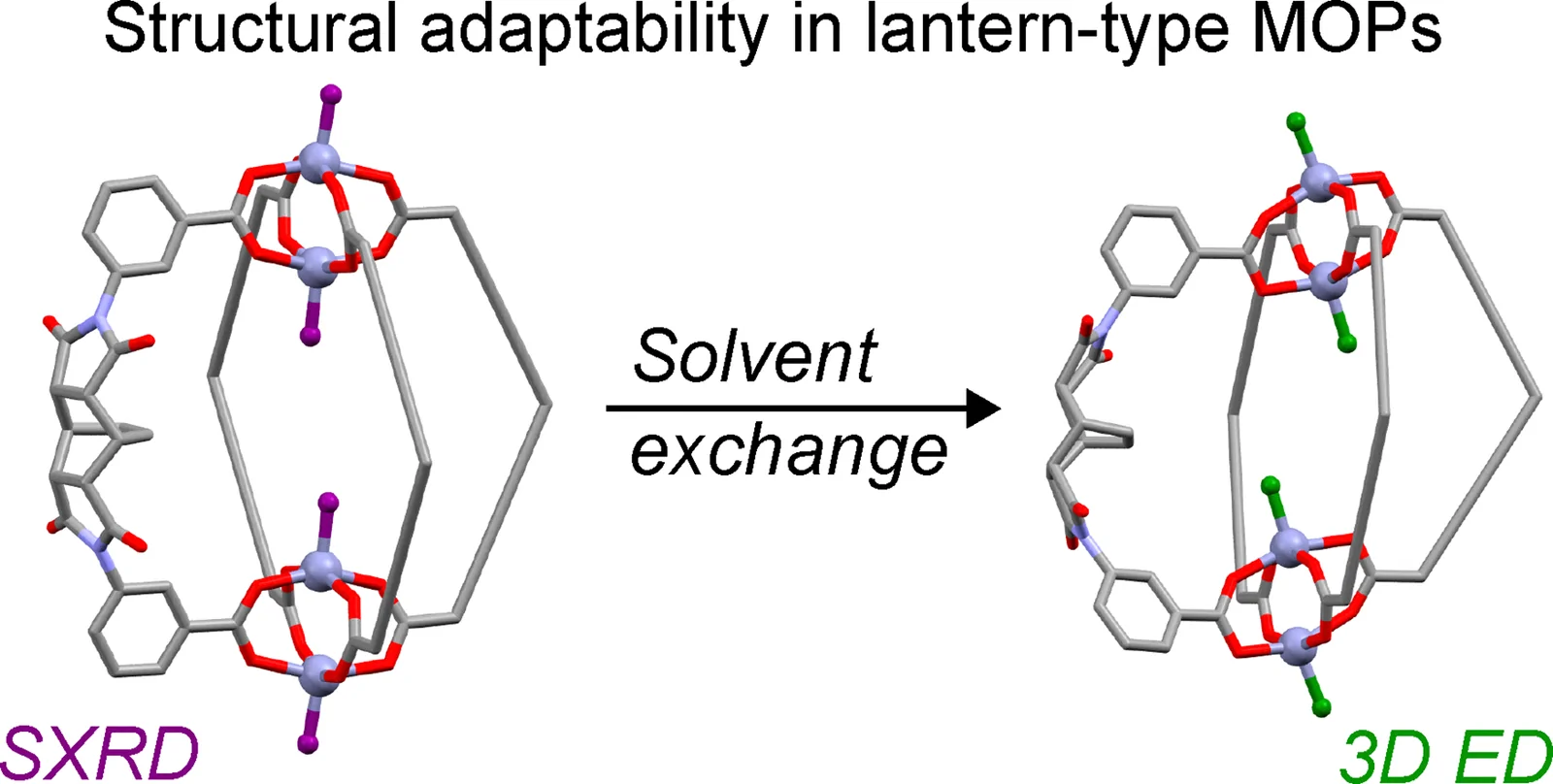

One of the pivotal advances in the development of metal–organic frameworks (MOFs) was the use of paddlewheel-based metal nodes. In terms of functionality, these nodes improved the stability of early frameworks to activation, facilitating the observation of permanent porosity in MOFs.^1–3^ From a chemical design standpoint, their use was an important stage in the continued development of strategies for the targeted synthesis of frameworks – reticular chemistry.^4,5^ Metal–organic polyhedra (MOPs) are the molecular analogues of MOFs.^6,7^ In the same way that the topology of MOFs can be designed by applying the principles of reticular chemistry to the synthetic building blocks, the geometry of the MOP can be targeted by considering the relative spatial disposition of binding groups in the ligand and the nature of the metal node.^8,9^ The combination of relatively rigid ligands containing carboxylic acid groups and metal ions that can be assembled into binuclear nodes has led to extensive families of MOPs including lantern ([M_4_L_4_]),^10–13^ octahedral ([M_12_L_12_]),^14–18^ and cuboctahedral ([M_24_L_24_])^19–22^ geometries. The reported paddlewheels for these cages include [Cr_2_],^23,24^ [Co_2_],^25^ [Ni_2_],^25,26^ [Cu_2_],^27–29^ [Mo_2_],^30–32^ [Ru_2_],^33–35^ and [Rh_2_];^36–38^ and heterometallic [MPd], where M = Ni, Cu, or Zn.^39,40^

[Zn_2_] paddlewheels have been prominent in the synthesis of MOFs,^1,41–45^ but they have yet to be reported in MOPs. In fact, a search of the CCDC showed that there are no confirmed molecular structures containing multiple [Zn_2_] paddlewheels. To target the formation of MOPs containing [Zn_2_] paddlewheels, we reacted the ligands MeOLH_2_, CH_3_LH_2_, and BrLH_2_, which contain rigid bicyclo[2.2.2]oct-7-ene units and had been used previously to successfully obtain Cu MOPs,^46^ with zinc(ii) acetate (synthetic details are provided in the SI). This approach yielded three new MOPs, and a 1D coordination polymer containing CH_3_LH_2_. The MOPs were found to be soluble in dimethylsulfoxide, facilitating their characterisation using NMR spectroscopy (Fig. S1–S3). The crystal structure of this coordination polymer, CH_3_-CP, showed its composition to be [Zn_2_(CH_3_L)_2_(DMA)_2_]_*n*_·2DMA where two CH_3_L^2−^ ligands form loops by bridging [Zn_2_] paddlewheels (Fig. S4). However, we struggled to reproduce this material in high yields for further study, and only its structure, IR, and PXRD data are presented in the SI.

The obtained lantern-type MOPs have the general formula [Zn_4_(RL)_4_(S)_4_]·*x*(solv) where the solvent content S and solv vary for each MOP, and the structures were confirmed using single crystal X-ray diffraction. The MOPs [Zn_4_(MeOL)_4_(DMF)_2_(H_2_O)_2_]·5.2DMF·1.8MeOH (MeO-MOP_DMF_) and [Zn_4_(BrL)_4_(H_2_O)_4_]·6.8DMA·0.5MeOH (Br-MOP_DMA_) crystallise in the triclinic space group *P*1̄, while [Zn_4_(CH_3_L)_4_(DMA)_2_(MeOH)_2_]·6.6DMA·3.2MeOH (CH_3_-MOP_DMA_) crystallises in the monoclinic space group *P*2_1_/*n*. All three molecules display the typical lantern geometry, with Br-MOP_DMA_ shown as an example in Fig. 1: two paddlewheels are connected by four deprotonated ligands to define the intrinsic pore of the cage (MeO-MOP_DMF_ and CH_3_-MOP_DMA_ are shown in Fig. S5). In each cage, the intrinsic pore sits on an inversion centre so that there are one and two crystallographically independent paddlewheels and ligands, respectively, in the structures. A notable distortion in the paddlewheel units containing Zn(ii) ions with respect to Cu(ii) is observed. The inner and outer metal ions of the paddlewheel sit further above and below the plane defined by the coordinating carboxylate ligands, resulting in an increased distance between the ions of the paddlewheel (Fig. 1). Previous computational studies of [Zn_2_] and [Cu_2_] paddlewheels found that this distortion arises from a combination of lower normal vibrational modes in the Zn(ii) paddlewheels together with an absence of the orbital directing effects that arise for the d^9^ Cu(ii) ion.^47^ All of the Zn(ii)-based cages show variation of the metric parameters of the cage in the as-synthesised phase with respect to the Cu(ii)-based materials, reflected in a variation in the distances between the interior metal ions of the MOPs (Zn *vs.* Cu: 8.649(1) *vs.* 8.435(1) Å, MeO-MOP; 8.371(1) *vs.* 8.157(1) Å, Br-MOP; 8.927(1) *vs.* 9.310(1) Å, CH_3_-MOP). The precise solvent content in each structure varies, meaning the alteration in these distances cannot be attributed solely to the change in metal ion, but the range of distances is indicative of an adaptability in the ligand skeleton.

**Fig. 1:**
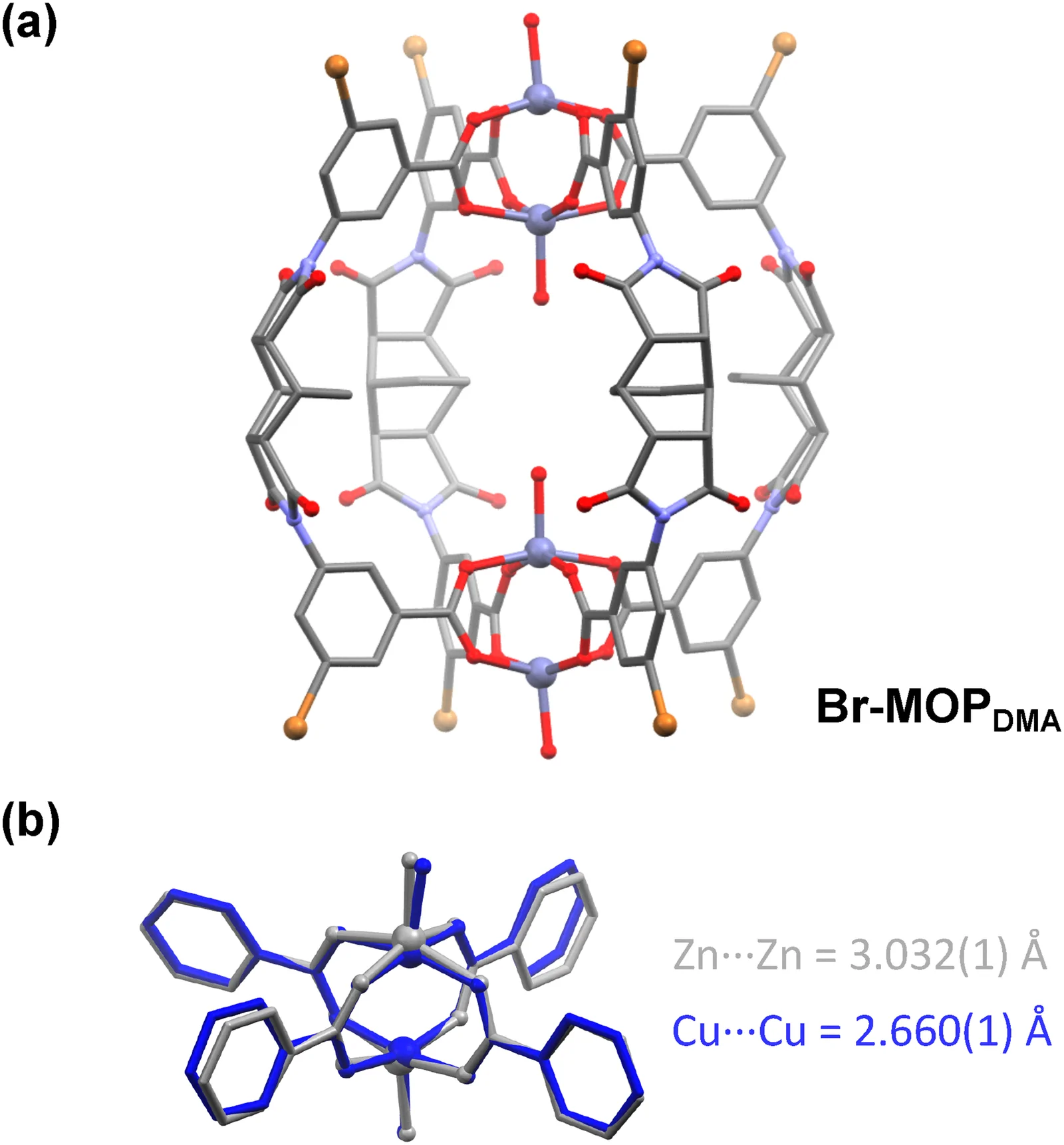
(a) The crystal structure of the cage unit [Zn_4_(BrL)_4_(H_2_O)_4_] in Br-MOP_DMA_. Hydrogen atoms and non-coordinated solvent molecules are omitted for clarity. (b) A structural overlay of the paddlewheel in Br-MOP_DMA_ with the Cu(ii) paddlewheel MOP reported previously (CSD REFCODE: ZIGFUU) showing the greater distortion in the Zn_2_ paddlewheel and greater distance between the metal ions.

The three MOPs were then solvent exchanged with MeOH in preparation for gas sorption measurements. For the resulting phases Br-MOP_MeOH_ and MeO-MOP_MeOH_, there is little change in the fingerprint region of the IR spectra below 1000 cm^−1^ with respect to the same region for the initial, as-synthesised solvates. In contrast, the bands in this region for CH_3_-MOP_MeOH_ show a marked resemblance to the data collected for CH_3_-CP (Fig. 2 and Fig. S6–S8). This leads us to suggest that the materials containing the ligands BrL^2−^ and MeOL^2−^ retain a discrete molecular structure through the solvent exchange process, while the discrete cage CH_3_-MOP is transformed into a polymeric phase. Although this type of solvent exchange process is commonplace in the study of MOPs, direct structural information of the resulting phases is not. The three resulting samples here all displayed: (i) a high degree of crystallinity and (ii) a diffractogram that was completely different to that of the as-synthesised solvates (the comparison for the MeO-MOP samples is shown in Fig. 3; see also Fig. S9–S11). Together with the reduction in the crystal size, this led us to use 3D electron diffraction (3D ED) to attempt to obtain the structures of the solvent exchanged phases.

**Fig. 2:**
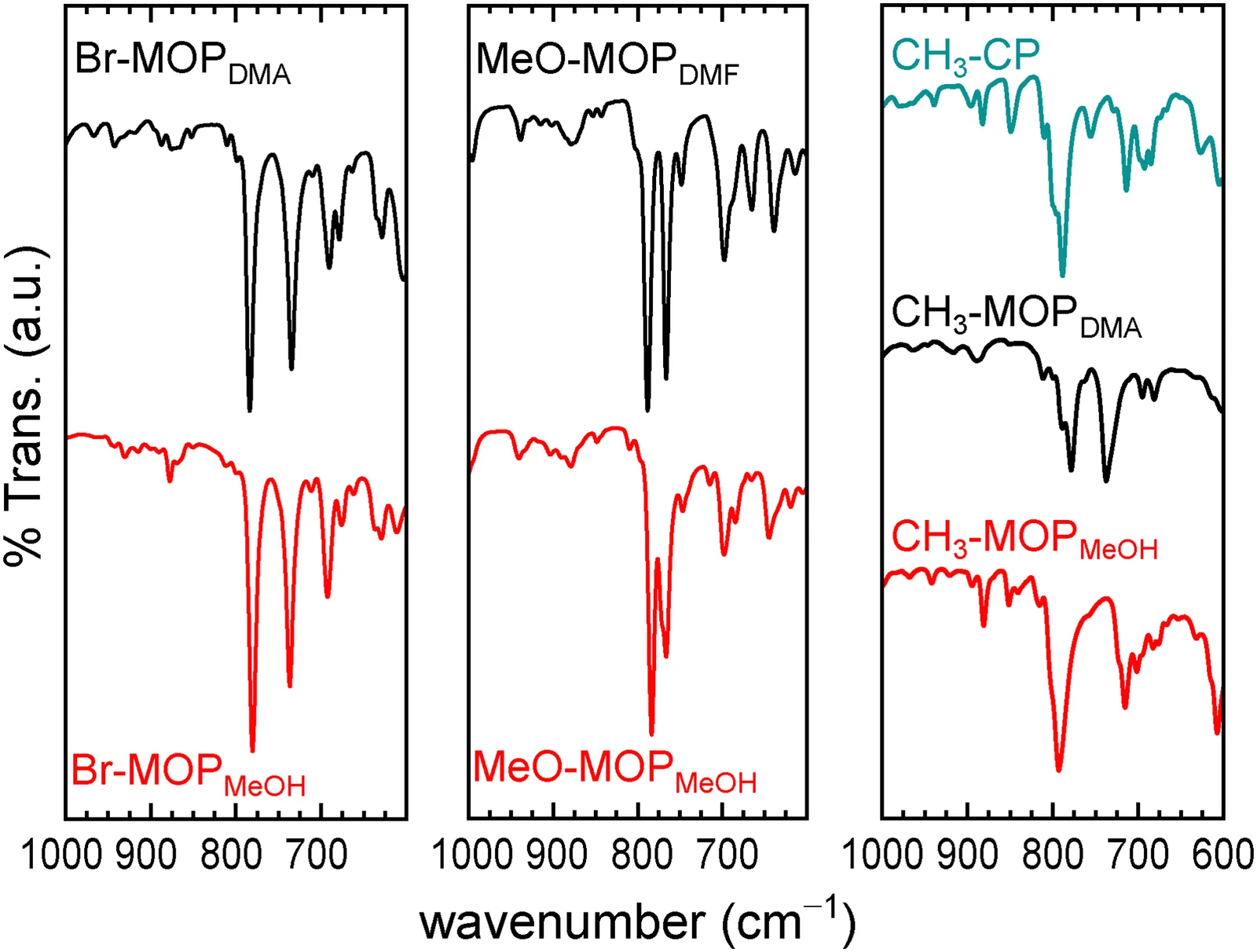
Comparison of the IR spectra collected for the three cages molecules in the as-synthesised phases, and after solvent exchange with MeOH.

**Fig. 3:**
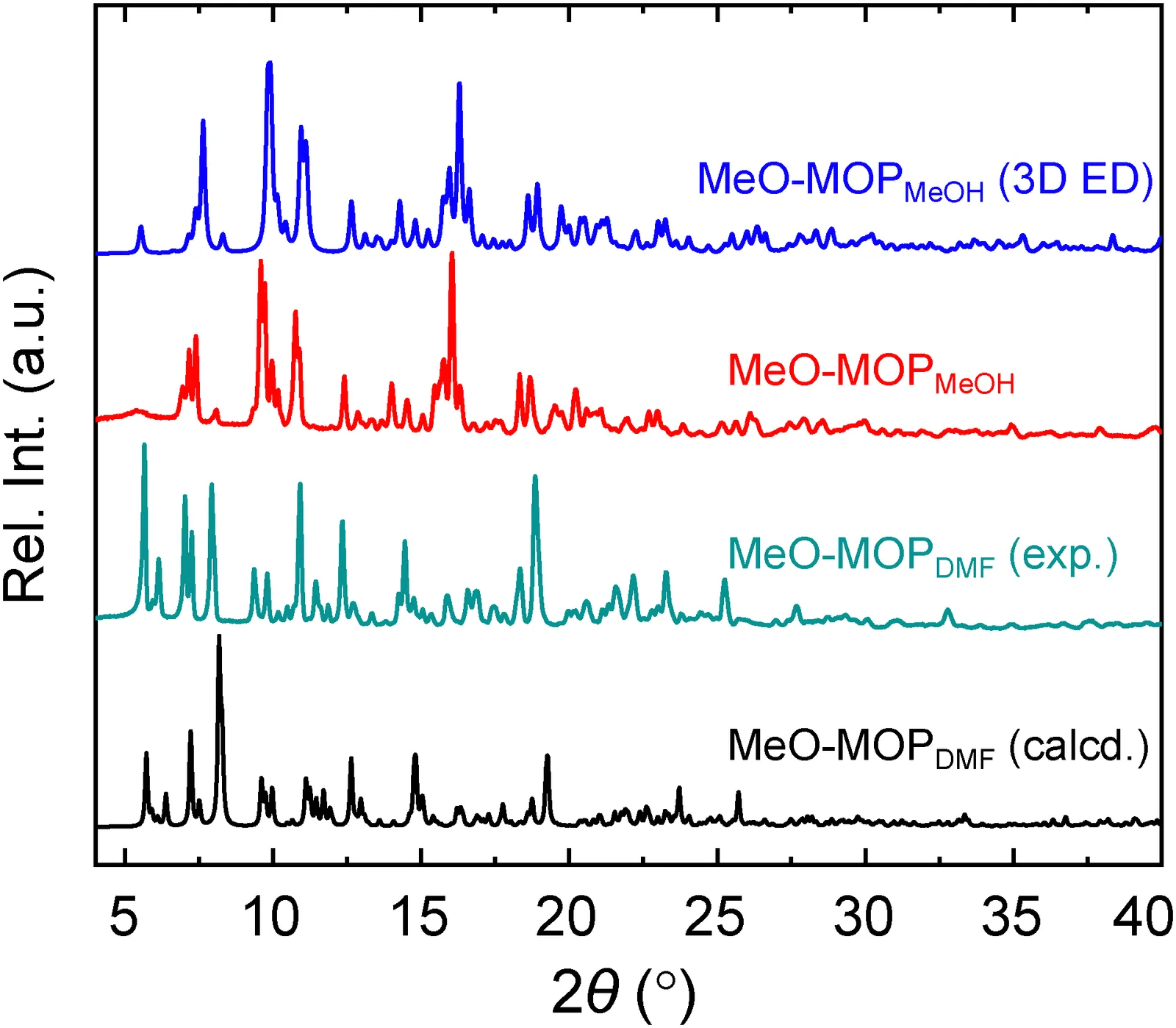
Comparison of the experimental powder X-ray diffraction data for the as-synthesised phase of the MOP MeO-MOP_DMF_ with the simulated diffractogram derived from the single crystal X-ray diffraction structure, and the analogous comparison for the solvent-exchanged phase MeO-MOP_MeOH_ and the simulated diffractogram from the 3D ED crystal structure.

Although 3D ED has proven to be a highly valuable tool in the study of framework materials,^48–51^ its use to study porous molecules such as MOPs has been more limited.^52,53^ In our recent study, we used it to show that solvent exchange in MOPs caused a structural transition from a discrete cage to a dense coordination polymer.^54^ The samples here were prepared from the dry powders of the solvent exchanged phases by dry dispersion onto TEM grids and loading into the Rigaku XtaLAB Synergy-ED electron diffractometer at 210 K, before the temperature was lowered for data collection (crystallographic parameters are compiled in Tables S2 and S3, both for the individual data collections and the merged data). We were successful in obtaining the structures of MeO-MOP_MeOH_ and Br-MOP_MeOH_. Both cages, MeO-MOP and Br-MOP, remain in the triclinic space group *P*1̄ after solvent exchange with MeOH, but with a large change in the dimensions of the respective unit cells reflected by a large reduction in volume: 3858.7(1) → 2908.7(3) Å^3^, for MeO-MOP_DMF_ → MeO-MOP_MeOH_; and 5260.7(2) → 2779.9(4) Å^3^, for Br-MOP_DMA_ → Br-MOP_MeOH_. This large change in volume for the Br-MOP cages is also reflective of the much lower degree of solvation in the solvent-exchanged phase for Br-MOP when compared to the as-synthesised phase.

The structure of Br-MOP_MeOH_ retains the same basic cage formula of [Zn_4_(BrL)_4_(H_2_O)_4_] as found in Br-MOP_DMA_, with disordered MeOH and water molecules filling both the internal cavity and the space between MOPs. For MeO-MOP_MeOH_, the cage structure shows that the coordinated DMF molecules from the initial as-synthesised phase have been replaced by water molecules, giving a formula for the MOP of [Zn_4_(MeOL)_4_(H_2_O)_4_]. Again, disordered water and MeOH molecules are found within and between the cages. Common to both structures is a compression of the cages with respect to the structures of the as-synthesised phases. This is represented by an overlay of the asymmetric units of these cages in Fig. 4. The distances between the internal Zn(ii) ions are reduced from 8.371(1) to 7.868(6) Å (Br-MOP_DMA_ → Br-MOP_MeOH_); and 8.649(1) to 8.101(4) Å (MeO-MOP_DMF_ → MeO-MOP_MeOH_). This compression is accommodated by the rotational flexibility in the ligand around the C–C bond between the carboxylic acid and phenyl ring; and dicarboximide N–C_benzoic acid_ bond. This solvent-driven compression is comparable to the type of breathing effects that are found in MOFs,^55^ with the difference being that cage compression is observed here in a discrete molecule, rather than being propagated throughout a network structure. The initial crystalline phases of the materials could be recovered by taking the MeOH solvated phases of MeO-MOP, Br-MOP, and CH_3_-MOP and re-soaking in DMF or DMA (Fig. S12–S14), although the recovered material is found to be less crystalline than when freshly synthesised.

**Fig. 4:**
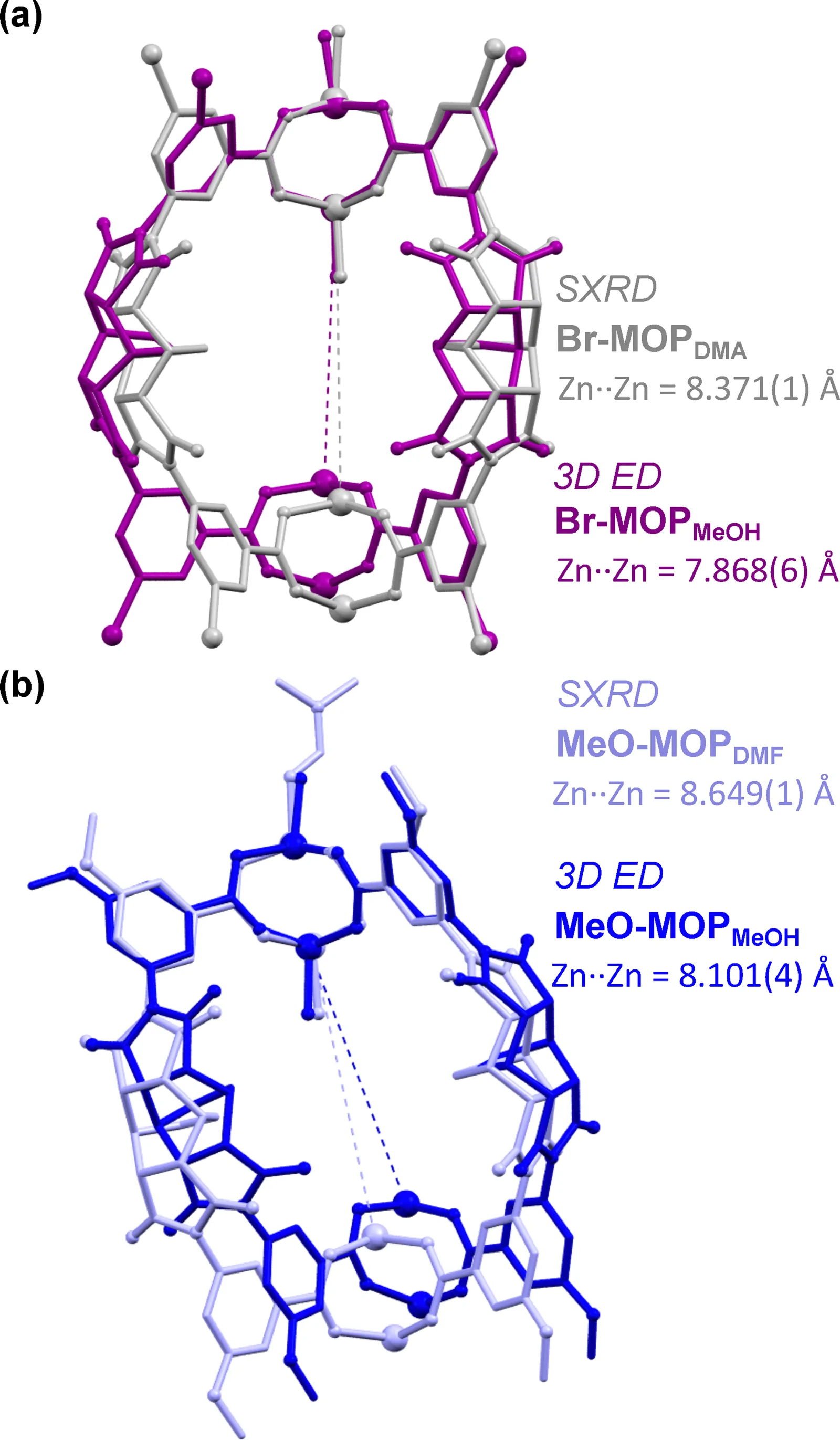
Overlay of the asymmetric units from the cages (a) Br-MOP_DMA_ and (b) MeO-MOP_DMF_ with the same from the solvent-exchanged cages Br-MOP_MeOH_ and MeO-MOP_MeOH_. The distances between the internal metal ions are shown in colours corresponding to the structural data. Hydrogen atoms and non-coordinated solvent molecules are omitted for clarity.

The gas sorption properties of all of these materials were then studied using N_2_ at 77 K and CO_2_ at 273 K, after activation under vacuum at 393 K. All three materials show comparable N_2_ sorption isotherms: low uptake is observed until around *P*/*P*_0_ ≥ 0.9, where the gas is an increase in sorption is observed due to bulk condensation (Fig. S15–S17). The observed CO_2_ adsorption isotherms are also comparable (Fig. 5), with modest uptakes attained at around 100 kPa. These uptakes are all significantly lower than those observed for the cages containing the [Cu_2_] paddlewheels. The IR spectra of the materials collected after gas sorption experiments were essentially the same as those collected prior to the measurements (Fig. S6–S8), suggesting that there is no further change in the connectivity of the materials after activation. However, the post-sorption PXRD data (Fig. S9–S11) evidence a loss of crystallinity in all of the materials. It may be that the ordered packing of the materials is unstable to vacuum, which could explain the low gas uptake observed, as well as why attempts to obtain the activated structures using 3D ED were unsuccessful, despite subjecting the cages to vacuum at lower temperatures than used in activation for gas sorption measurements. However, it may also be the case that this loss of crystallinity is induced by gas sorption, rather than the activation process. The crystallinity of the materials can be recovered by soaking in MeOH (Fig. S12–S14).

**Fig. 5:**
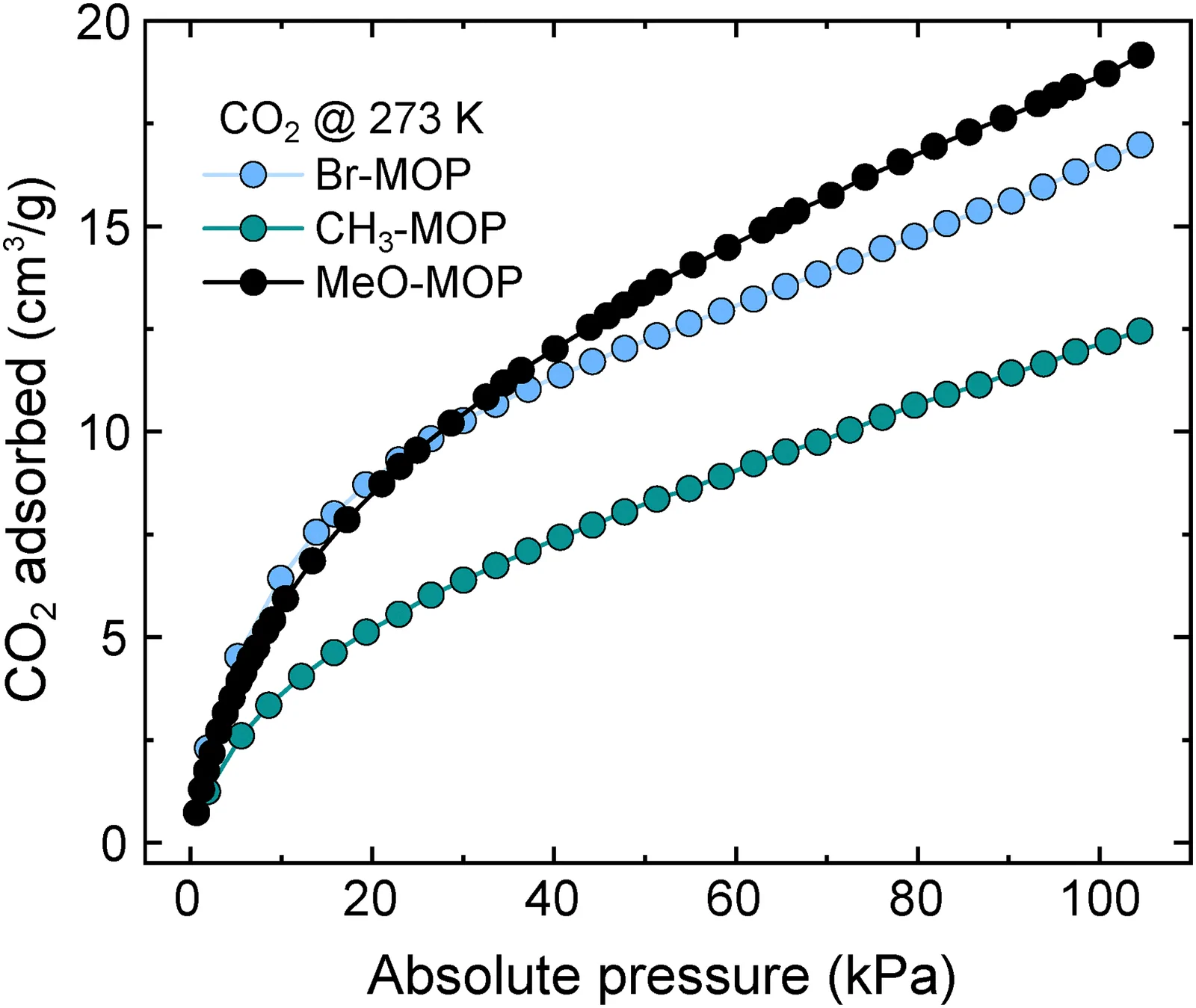
Gas sorption isotherms measured at 273 K for the three materials Br-MOP, CH_3_-MOP, and MeO-MOP after activation under vacuum at 393 K.

In conclusion, we have presented a family of [Zn_2_] paddlewheel-based MOPs, which are among the first of their kind. The solvent exchange processes that the cages were subjected to caused reductions in crystal size that previously would have rendered structure solution impossible. We were able to address this issue for some of the cages using 3D ED, which revealed a structural flexibility in the MOPs. There are still limitations to this approach – we were not able to obtain 3D ED data for all of the compounds we report, related to challenges in sample preparation – but the work contributes to a growing number of reports using 3D ED to study porous molecules, and proves its ability to give insight that would not otherwise have been possible.

## Author contributions

MPS, BDS, MGW, and ERM performed all synthetic procedures and collected IR, NMR, SXRD, PXRD, and N_2_ and CO_2_ sorption data, as well as carrying out initial data interpretation. CSS, JPT, and SP collected and interpreted the 3D electron diffraction data. AJF supervised the interpretation of the gas sorption data. GAC conceived and supervised the project. All authors discussed the results and commented on the drafts of the manuscript written by MPS and GAC.

## Conflicts of interest

There are no conflicts to declare.

## Supplementary Material

CC-062-D6CC04492A-s001

CC-062-D6CC04492A-s002

## Data Availability

The raw data that support the findings of this study are openly available from the University of Strathclyde KnowledgeBase at https://doi.org/10.15129/68155775-bc0c-4573-8943-09b624bf7e71. Supplementary information (SI) is available, and contains synthetic and physical characterisation procedures, and additional NMR and IR spectra; diffraction data; and gas sorption data. See DOI: https://doi.org/10.1039/d6cc04492a. CCDC 2560137–2560140, 2471726 and 2471727 contain the supplementary crystallographic data for this paper.^56*a*–*f*^
