# Supplementary material for: 3D electron diffraction shows structural flexibility in rare Zn(ii) paddlewheel-based metal–organic polyhedra
Source: Chem Commun (Camb). 2026 Aug 12;62(72):17898–902. doi: 10.1039/d6cc04492a (PMC13576399; doi:10.1039/d6cc04492a)
Supplement: Supplementary file 1 [file CC-062-D6CC04492A-s001.pdf]

Supplementary Information (SI) for ChemComm.
This journal is © The Royal Society of Chemistry 2026

Supporting information for "3D electron diffraction shows structural flexibility in rare Zn(II) paddlewheel-based metal-organic polyhedra"

Matthew P. Snelgrove,[a] Beatriz Doñagueda Suso,[a] Calum S. Sangster,[b] Megan G. Wilkinson,[a] Emma Regincos Marti,[a] Jeremiah P. Tidey,[c] Alan R. Kennedy,[a] Simon Parsons,[b] Ashleigh J. Fletcher[d] and Gavin A. Craig[a]*

[a]*Department of Pure and Applied Chemistry, University of Strathclyde, Glasgow, United Kingdom, G1 1RX.* gavin.craig@strath.ac.uk.

[b]*EaStCHEM, School of Chemistry and Centre for Science at Extreme Conditions, University of Edinburgh, Edinburgh, United Kingdom, EH9 3FJ.*

[c]*Department of Physics, University of Warwick, Gibbet Hill Road, Coventry, United Kingdom, CV4 7AL.*

[d]*Department of Chemical and Process Engineering, University of Strathclyde, Glasgow, United Kingdom, G1 1XJ.*

Experimental details

Synthesis

All reagents were used as purchased without further purification. The ligands **MeOLH$_2$**, **BrLH$_2$**, and **MeLH$_2$** were synthesised following a previously reported procedure.[1]

[Zn$_4$(MeOL)$_4$(DMF)$_2$(H$_2$O)$_2$]·5.2DMF·1.8MeOH. (**MeO-MOP$_{DMF}$**) For single-crystals: a solution of MeOLH$_2$ (160 mg, 0.30 mmol) in DMF (1 mL) and lutidine (3 drops) was combined with a solution of Zn(OAc)$_2$·2H$_2$O (64 mg, 0.29 mmol) in DMF (1 mL). The resulting solution was placed in an oven at 120 °C for 3 days. The resulting amber solution was split in two pipettes and layered with MeOH, giving colourless crystals suitable for single crystal x–ray diffraction. Only one of the pipettes was filtered, giving 18 mg of colourless crystals (Yield: 14%). For the bulk sample: MeOLH$_2$ (0.500 g, 0.92 mmol, 1 eqs.) was dissolved in DMF (2.5 mL) and added to a solution of Zn(OAc)$_2$·2H$_2$O (0.200 g, 0.92 mmol, 1 eqs.), also in DMF (2.5 mL). To this, lutidine (210 µL, 1.83 mmol, 2 eqs.) was added to the solution and left to stand overnight. At this point, a white precipitate formed that was collected *via* vacuum filtration and washed with DMF to give the named compound (0.517 g, 77% yield based on crystallographic formula). Elemental analysis: Found (C%, H%, N%) 51.3, 4.9, 7.8; Calc. (For [Zn$_4$(MeOL)$_4$(DMF)$_2$(H$_2$O)$_2$]·8DMF·6H$_2$O, C%, H%, N%) 51.5, 5.1, 7.6. The IR and PXRD data presented correspond to this bulk sample. **MeO-MOP$_{MeOH}$** was obtained by soaking this material in MeOH, and exchanging the supernatant for fresh MeOH for a total of seven exchanges. NMR spectra for both phases shown in Fig. S1.

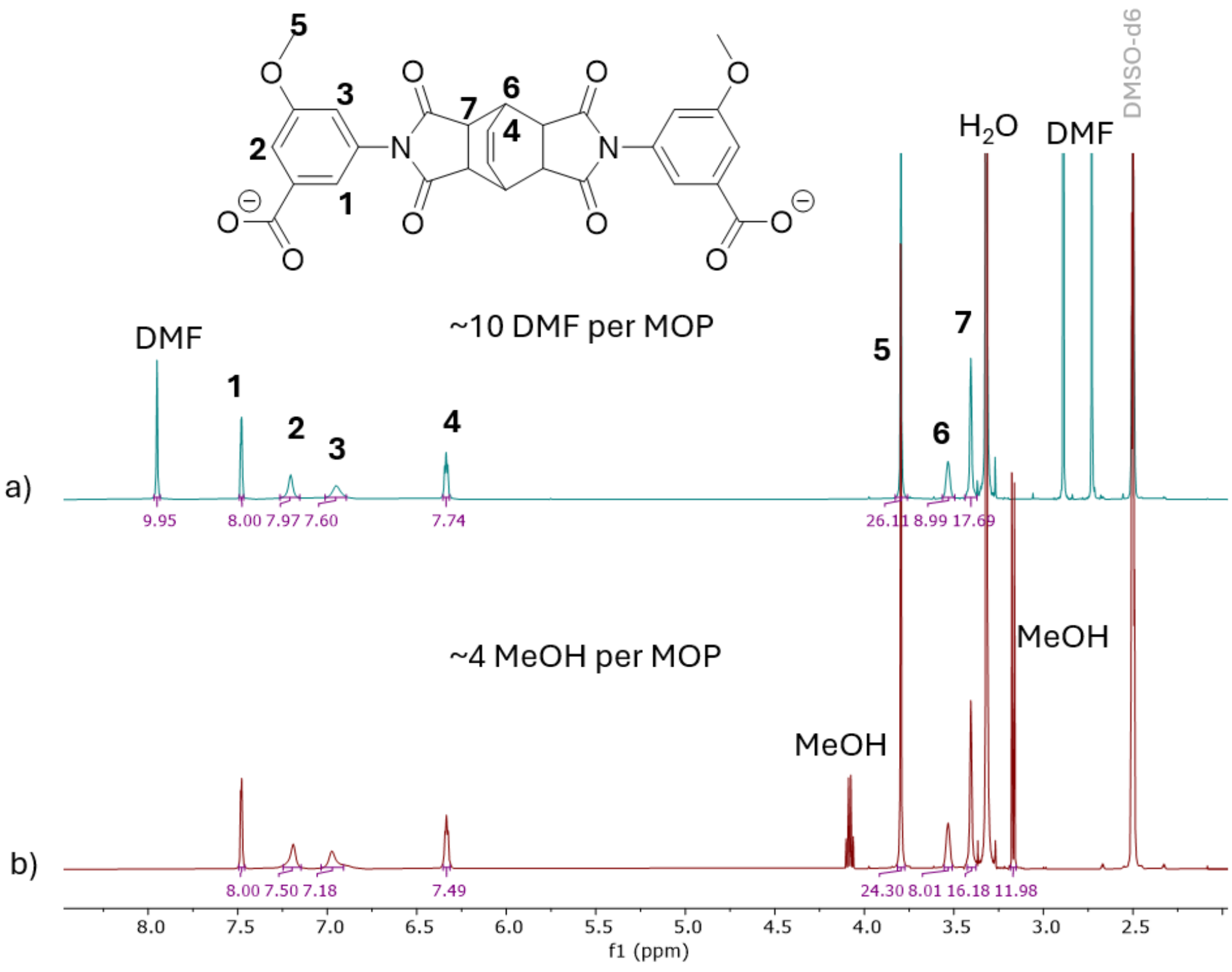

Figure S1. ^{1}H NMR spectra in dmso-d_6 for (a) **MeO-MOP$_{DMF}$** and (b) **MeO-MOP$_{MeOH}$**.

[$Zn_4(CH_3L)_4(DMA)_2(MeOH)_2$]·6.6DMA·3.2MeOH. (**$CH_3$-MOP$_{DMA}$**) CH_3LH_2 (0.500 g, 0.970 mmol, 1 eqs.) was dissolved in N,N-dimethylacetamide (3.75 mL) and added to a solution of zinc(II) acetate dihydrate (0.216 g, 0.970 mmol, 1 eqs.) in N,N-dimethylacetamide (3.75 mL). To this, 2,6-lutidine (195 µL, 1.94 mmol, 2 eqs.) was added to the solution and carefully layered with MeOH. Over the course of 3 days, a white precipitate and crystals formed simultaneously, and were collected *via* vacuum filtration and washed with copious amounts of DMA to give the named compound (0.537 g, 77% yield based on crystallographic formula). Manual separation of the crystals and powder, followed by IR spectroscopy and powder X-ray diffraction showed these phases to be the same material. Elemental analysis: Found (C%, H%, N%) 54.7, 5.7, 8.2; Calc. (For [$Zn_4(CH_3L)_4(DMA)_2(MeOH)_2$]·9DMA·6$H_2O$, C%, H%, N%) 55.1, 5.8, 7.7. The IR and PXRD data presented correspond to the combined crystals and powder. **CH_3-MOP$_{MeOH}$** was obtained by soaking this material in MeOH, and exchanging the supernatant for fresh MeOH for a total of seven exchanges. NMR spectra for both phases shown in Fig. S2.

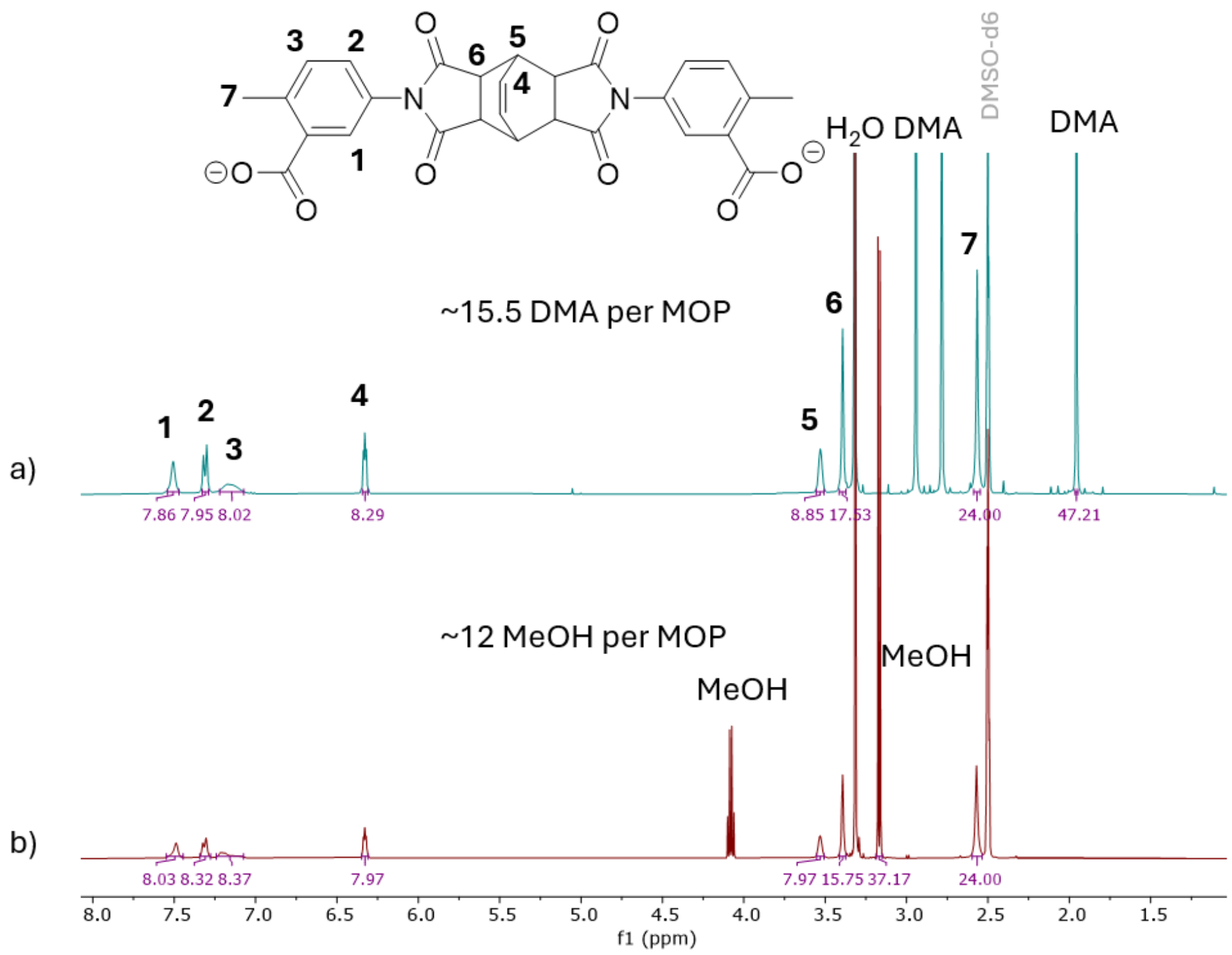

Figure S2. ^{1}H NMR spectra in dmso-d_6 for (a) **CH_3-MOP$_{DMA}$** and (b) **CH_3-MOP$_{MeOH}$**.

[$Zn_4(CH_3L)_2(DMA)_4$]$_n$·2DMA (CH_3-CP). A solution of CH_3LH_2 (152 mg, 0.30 mmol) in DMA (1 mL) and lutidine (3 drops) was combined with a solution of $Zn(OAc)_2·2H_2O$ (65 mg, 0.30 mmol) in DMA (1 mL). The resulting solution was placed in an oven at 120 °C for 3 days, giving 66 mg of colourless crystals suitable for single crystal X–ray diffraction, (Yield: 48.2%). This material can also be synthesised in extremely low yield by following the synthesis for **CH_3-MOP$_{DMA}$** but not layering with MeOH.

$[Zn_4(BrL)_4(H_2O)_4]\cdot 6.8DMA\cdot 0.5MeOH$. (Br-MOP$_{DMA}$). $BrLH_2$ (0.500 g, 0.776 mmol, 1 eqs.) was dissolved in N,N-dimethylacetamide (2 mL) and added to a solution of zinc(II) acetate dihydrate (0.170 g, 0.776 mmol, 1 eqs.) in N,N-dimethylacetamide (2 mL). To this, 2,6-lutidine (180 µL, 1.55 mmol, 2 eqs.) was added to the solution and left to stand for 3 days. At this point, large colourless crystals had formed which were collected *via* vacuum filtration and washed with copious amounts of DMA to give the named compound (0.207 g, 52% yield based off crystallographic formula). Elemental analysis: Found (C%, H%, N%) 45.8, 4.9, 7.7; Calc. (For $[Zn_4(BrL)_4(H_2O)_4]\cdot 14DMA\cdot 3H_2O$, C%, H%, N%) 46.0, 4.7, 7.4. **Br-MOP$_{MeOH}$** was obtained by soaking this material in MeOH, and exchanging the supernatant for fresh MeOH for a total of seven exchanges. NMR spectra for both phases shown in Fig. S3.

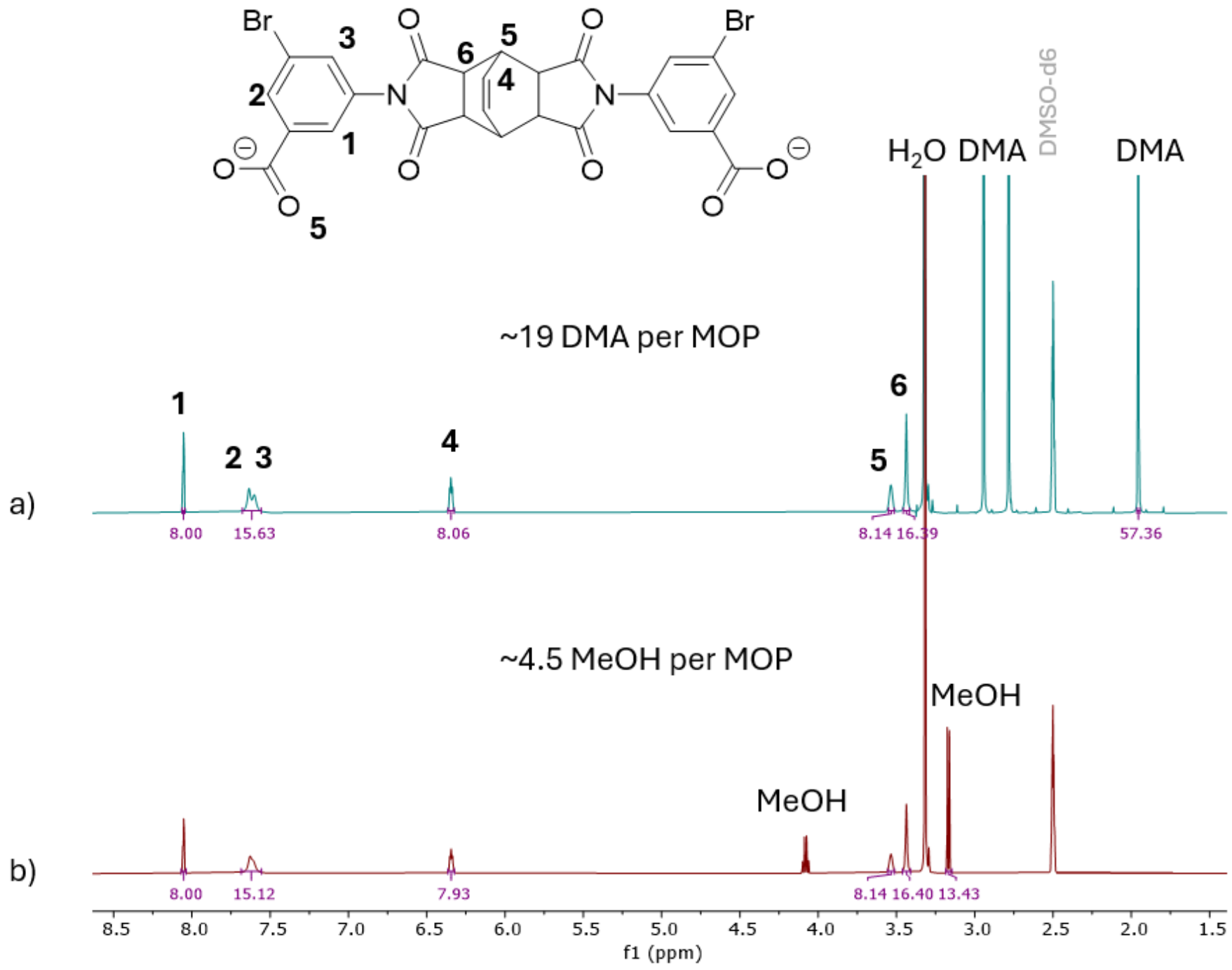

Figure S3. ^{1}H NMR spectra in dmso-d_6 for (a) **Br-MOP$_{DMA}$** and (b) **Br-MOP$_{MeOH}$**.

Physical characterisation

NMR data were collected at room temperature on a Bruker AVANCE 400 NMR spectrometer, operating at 400.13 MHz for ^{1}H. Elemental analyses were collected using a Thermo Scientific FlashSmart CHNS Elemental Analyzer in the Department of Civil and Environmental Engineering at the University of Strathclyde. Powder X-Ray Diffraction (PXRD) data were collected at room temperature over a flat plate using a poly(methyl methacrylate) holder. The instrument used was a Bruker D8 Discover in a $\vartheta/2\vartheta$ Bragg-Brentano reflection geometry using a Cu K_α source (λ = 1.54056 Å), available in the Centre for Continuous Manufacturing and Advanced Crystallisation (CMAC), at the University of Strathclyde. Fourier-Transformed infra-red spectra were collected using a NICOLET iS5 Thermo Scientific spectrometer, with parameters set as 48 scans and a resolution of 4 cm^{-1}. Prior to gas sorption measurements, samples were activated *in situ* by heating at 120 °C for 10 hours under vacuum. N_2 uptake was measured at −196 °C using a Micromeritics ASAP 2420 (available

in the Department of Chemical and Process Engineering at the University of Strathclyde), using oxygen free nitrogen gas at pressures from 0 to 760 mmHg. CO_2 uptake was measured at 0 °C, using a Micrometrics ASAP 2425 (available in the Department of Chemical and Process Engineering, at the University of Strathclyde), using pressures in the range 0 to 760 mmHg.

Single crystal X-ray diffraction measurements.

Single-crystal X-ray diffraction data were measured with a Rigaku Synergy-i instrument with monochromated Cu−Kα (λ = 1.54184 Å) radiation. The measured data were processed with the CrysAlisPro[2] software package, solved with the ShelXT[3] structure solution program, and refined with ShelXL-2018[3] to convergence against F^2. Final refinement was within OLEX2 or WinGX.[4,5] Data for **CH_3-MOP$_{DMA}$** was treated as twinned by a 180° rotation about reciprocal [0.14 0 1] and the final refinement was against a hklf 5 formatted reflection file. The larger twin component refined to a 0.5701(11) contribution. Where possible solvent molecules within the cavities (including those that were disordered or on partially occupied sites) were modelled, even where only a relatively crude approximation was possible. However, some solvent sites in **MeO-MOP$_{DMF}$** and **Br-MOP$_{DMA}$** could not be sensibly modelled. The contributions of these components were removed using the SQUEEZE routine as implemented in PLATON.[6] This removed approximately 93 and 553 electron equivalents from 340 and 1946 Å^{3}of unit cell volume for **MeO-MOP$_{DMF}$** and **Br-MOP$_{DMA}$** respectively. Numerous moieties were refined as disordered over two sites. Where necessary appropriate restraints and constraints were added to these groups to ensure that geometric and displacement parameters approximated to normal behavior. Disordered groups included; a methoxy substituent and solvent molecules in **MeO-MOP$_{DMF}$**, solvent molecules in **CH_3-MOP$_{DMA}$**, both DMF ligands and DMF solvate molecules in **CH_3-CP**, and a C_6Br unit and the solvent molecules of **Br-MOP$_{DMA}$**.

Selected X-ray crystallographic and refinement data are given in Table S1. Full single crystal diffraction data for all structures reported in crystallographic information files (CIF) accompanying this document and deposited with the CCDC as deposition numbers 2560137 to 2560140. Full details on data collection, reduction and refinement can be found in the individual CIFs.

3D electron diffraction measurements.

Data Collection

For **MeO-MOP$_{MeOH}$** and **Br-MOP$_{MeOH}$**, sample was lightly ground between two glass slides and dispersed dry onto a copper-supported holey carbon TEM grids and loaded into the Rigaku XtaLAB Synergy-ED electron diffractometer using a Gatan Elsa model 689 cryo-holder. Both samples were held at 210 K in the sample chamber to sublime the ice before cooling to collection temperatures of (**MeO-MOP$_{MeOH}$**) 175 K and (**Br-MOP$_{MeOH}$**) 125 K. The use of different measurement temperatures was due to constraints on available

measurement time. The instrument was operated at 200 kV and equipped with a Rigaku HyPix-ED hybrid pixel array area detector.

In each case, data for a range of crystallites were surveyed using continuous rotation electron diffraction with a selected area of ~2 µm diameter at the image plane using CrysAlisPRO (version 1.171.44.79a for **MeO-MOP$_{MeOH}$**, 1.171.44.80a for **Br-MOP$_{MeOH}$**.[2] The reported data are subsequently taken from the merging of seven (**MeO-MOP$_{MeOH}$**) and four (**Br-MOP$_{MeOH}$**) datasets of comparable quality over tilt ranges between 73 and 122 ° from crystallites appearing as blocks with dimensions in the order of 0.3 – 1 µm and using frame scan widths of 0.25 ° in all cases. Datasets were in each case individually indexed and integrated, and subsequently merged and scaled together for each model using CrysAlisPRO (version 1.171.44.115a)[2] and no absorption corrections were applied.

Structure solution and refinement

The structures were solved using ShelXT[3] and refined in the kinematic approximation using Olex2.refine[7] as implemented in Olex2 (version 1.5-ac7-016, compiled 2025.05.29 svn.r8cd99b3d for Rigaku Oxford Diffraction, GUI svn.r7254)[4] using published scattering factors.[8] An extinction correction was applied in each case to broadly account for the impact of multiple diffraction with further omission of outlying reflections in the final stages of the refinement; the poor agreement was assumed to arise from either from multiple scattering effects, by being obscured during collection or by limitations in the automated outlier rejections applied during data reduction.

All chemically equivalent covalent bonds in each model were restrained to have similar distances. In all cases, C-H hydrogen atoms were placed at geometrically constrained positions at 'neutron' distances. H-atoms of water molecules were initially placed geometrically in such a way as to optimise H-bonding and refined as rigid bodies. The geometric constraints were subsequently removed and replaced by similarity restraints on O-H bond distances. H-atoms were omitted if this model proved to be unstable on refinement. Further explicit distance restraints were applied to the C-O bonds in MeOH solvate molecules and around the midpoint of one (D)H···A interaction in **Br-MOP$_{MeOH}$**.

All non-H atoms of the Zn complexes were modelled with anisotropic displacement parameters (ADPs) with enhanced rigid bond restraints applied globally.[9] In addition, the ADPs of C13A and C24A in **MeO-MOP$_{MeOH}$** were restrained to be approximately isotropic. Solvent molecules were modelled with a mixture of isotropic and anisotropic displacement parameters. The isotropic displacement parameters of hydrogen atoms were set equal to 1.5× or 1.8× the equivalent isotropic displacement parameters of the parent atoms. Complete experimental and refinement information are contained in the deposited CIFs along with structure factors and embedded .RES files. These are deposited in the CSD with CCDC reference codes CCDC 2471726 (**MeO-MOP$_{MeOH}$**) and 2471727 (**Br-MOP$_{MeOH}$**). Tables S2 and S3 report experimental parameters from the merged refinement and individual datasets for **MeO-MOP$_{MeOH}$** and **Br-MOP$_{MeOH}$**, respectively.

Table S1. Selected crystallographic data for the single crystal X-ray diffraction structures.

	**MeO-MOP$_{DMF}$**	**CH_3-MOP$_{DMA}$**	**Me-CP**	**Br-MOP$_{DMA}$**
λ (Å)	1.54184			
CCDC ref no.	2560137	2560138	2560139	2560140
Crystal System	Triclinic	Monoclinic	Monoclinic	Triclinic
Space Group	P-1	$P2_1/n$	$P2_1/n$	P-1
T(K)	100(2)	150(2)	100(2)	100(2)
a (Å)	15.6796(2)	14.9219(2)	10.6886(1)	17.2898(3)
b (Å)	16.7157(2)	19.0317(2)	38.9126(3)	19.5483(4)
c (Å)	17.1271(2)	30.5510(2)	16.8834(3)	19.6117(3)
α (°)	64.348(1)	90	90	62.076(2)
β (°)	83.123(1)	103.302(1)	94.392(1)	64.116(2)
γ (°)	72.490(1)	90	90	80.833(2)
V (Å^3)	3858.72(9)	8443.38(16)	7001.54(15)	5260.7(2)
Z	1	2	8	1
$2\vartheta_{max}$ (°)	147.326	144.706	148.306	147.612
Reflections	80941	139344*	140154	104083
Unique Data	15369	28995*	13942	20906
Observed Data	13437	22675*	13178	16739
No. Parameters	969	1039	1043	949
R_{int}	0.0454	0.0577*	0.0569	0.0765
$R[F^2 > 2\sigma(F^2)]$	0.0721	0.0738	0.0369	0.0761
$wR(F^2)$	0.2384	0.2356	0.0976	0.2525
S	1.058	1.029	1.041	1.083
ρ_{max}, ρ_{min} (eÅ^{-3})	1.607/−0.931	1.352/-0.626	0.596/−0.476	1.450/−1.427

* Processed as a twin.

Table S2. Selected crystallographic data for the 3D ED structure of **MeO-MOP$_{MeOH}$**.

**Merged Crystal Data**	
CCDC ref no.	2471726
Chemical formula	$C_{112}H_{88}N_8O_{44}Zn_4 \cdot 5.469(H_2O) \cdot 2(O) \cdot 1.469(CH_4O)$
M_r	2689.15
Crystal system, space group	Triclinic, *P*-1
Temperature (K)	175
a, *b*, *c* (Å)	12.9567(7), 14.4222(7), 17.4506(8)
α, β, γ (°)	104.292(4), 105.134(5), 102.548(5)
V (Å^3)	2908.7(3)
Z	1
Radiation type	Electron, λ = 0.02510 Å
No. Crystals	8
**Merged dataset**	
No. reflections (meas., indep., obs [$I \geq 2u(I)$])	80896, 11569, 7749
R_{int}	0.294
$(\sin \theta/\lambda)_{max}$ (Å^{-1})	0.626
**Refinement**	
R_1, wR_2, [$F^2 > 2\sigma(F^2)$]	0.1801, 0.3459
R_1, wR_2, [all]	0.2318, 0.3731
GoF(*S*) (inc., exlc. restraints)	1.0167, 1.0557
No. of reflections	11569
No. of parameters	844
No. of restraints	839
$\Delta\phi_{max}$, $\Delta\phi_{min}$ (as output by Olex2.refine)	1.39, −1.17

Table S3. Selected crystallographic data for the 3D ED structure of **Br-MOP$_{MeOH}$**.

Merged Crystal Data	
CCDC ref no.	2471727
Chemical formula	$C_{104}H_{64}Br_8N_8O_{36}Zn_4 \cdot 4(H_2O) \cdot 1.316(CH_4O) \cdot 2.684(O)$
M_r	3059.71
Crystal system, space group	Triclinic, *P*−1
Temperature (K)	125
a, *b*, *c* (Å)	13.8777(9), 15.4439(11), 15.6658(9)
α, β, γ (°)	103.315(6), 109.119(6), 108.932(6)
V (Å^3)	2779.9(4)
Z	1
Radiation type	Electron, λ = 0.02510 Å
No. Crystals	7
**Merged dataset**	
No. reflections (meas., indep., obs [I ≥ 2u(I)])	66761, 9519, 6750
R_{int}	0.256
$(\sin \theta/\lambda)_{max}$ (Å^{-1})	0.598
**Refinement**	
R_1, wR_2, [$F^2 > 2\sigma(F^2)$]	0.1877, 0.3704
R_1, wR_2, [all]	0.2283, 0.3933
GoF(*S*) (inc., exlc. restraints)	1.0014, 1.0477
No. of reflections	9519
No. of parameters	791
No. of restraints	826
$\Delta\phi_{max}$, $\Delta\phi_{min}$ (as output by Olex2.refine)	1.41, −1.34

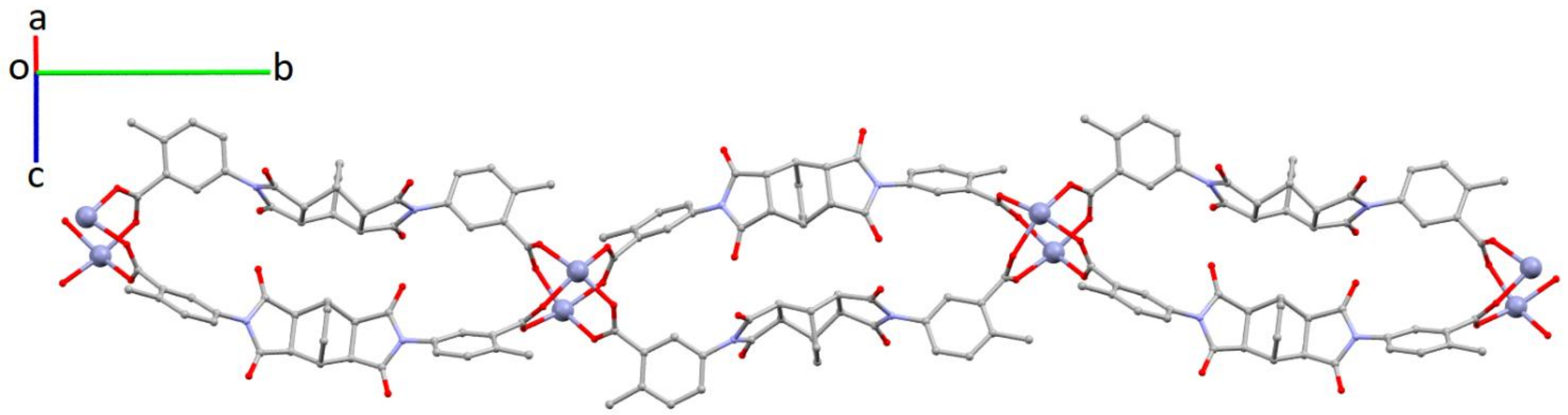

Figure S4. View of the 1D chains that are formed in **CH_3-CP** parallel to the *b*-axis of the crystal structure. DMA molecules that are coordinated to the axial sites of the paddlewheels have been omitted from clarity, as have hydrogen atoms and DMA molecules that are found in the voids of the crystal structure.

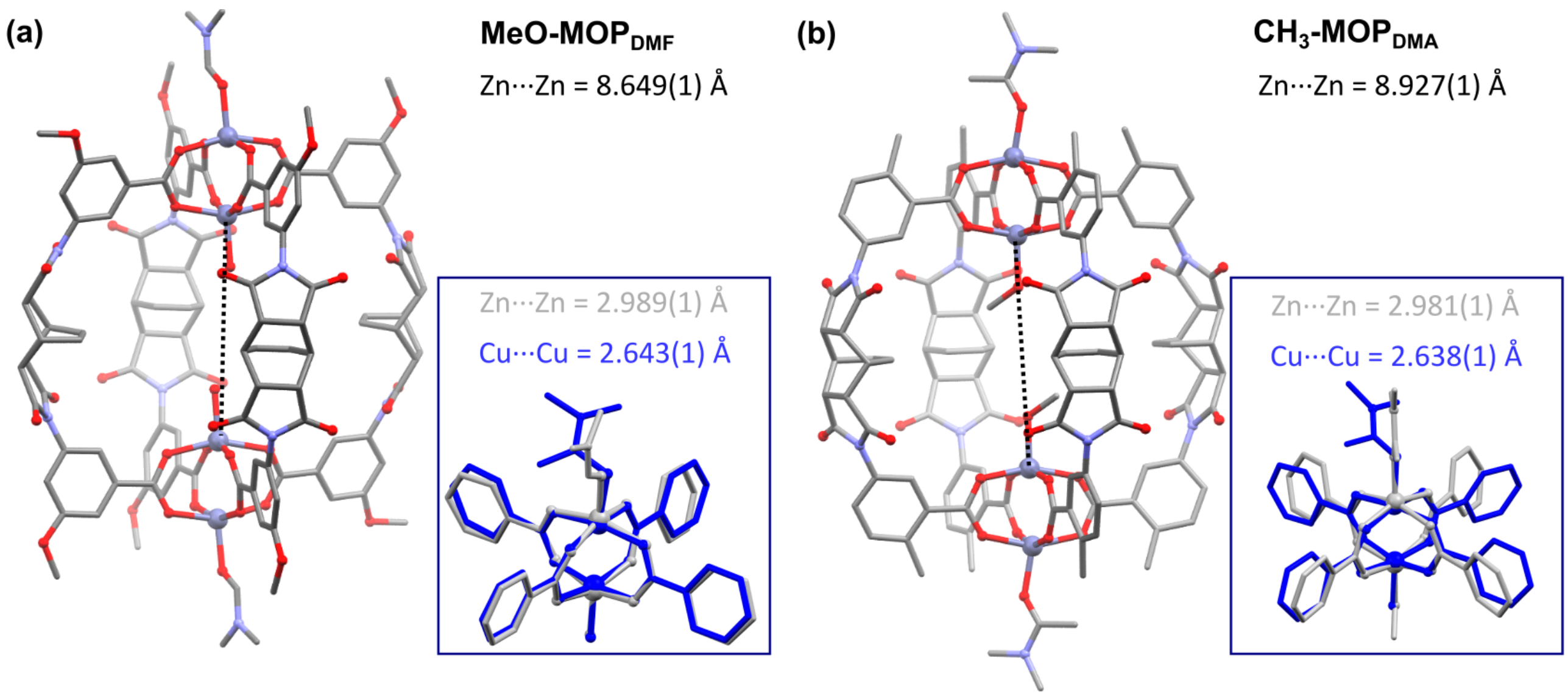

Fig. S5. View of the cages (a) **MeO-MOP$_{DMF}$** and (b) **CH_3-MOP$_{DMA}$**, with the distances given between the internal Zn(II) ions of the paddlewheel. Hydrogen atoms, non-coordinated solvent, and disorder in ligand moieties are omitted for clarity. The boxes show the overlay of the Zn paddlewheels in these cages with the previously reported Cu(II) paddlewheels, and give the distance between the ions within the paddlewheel.

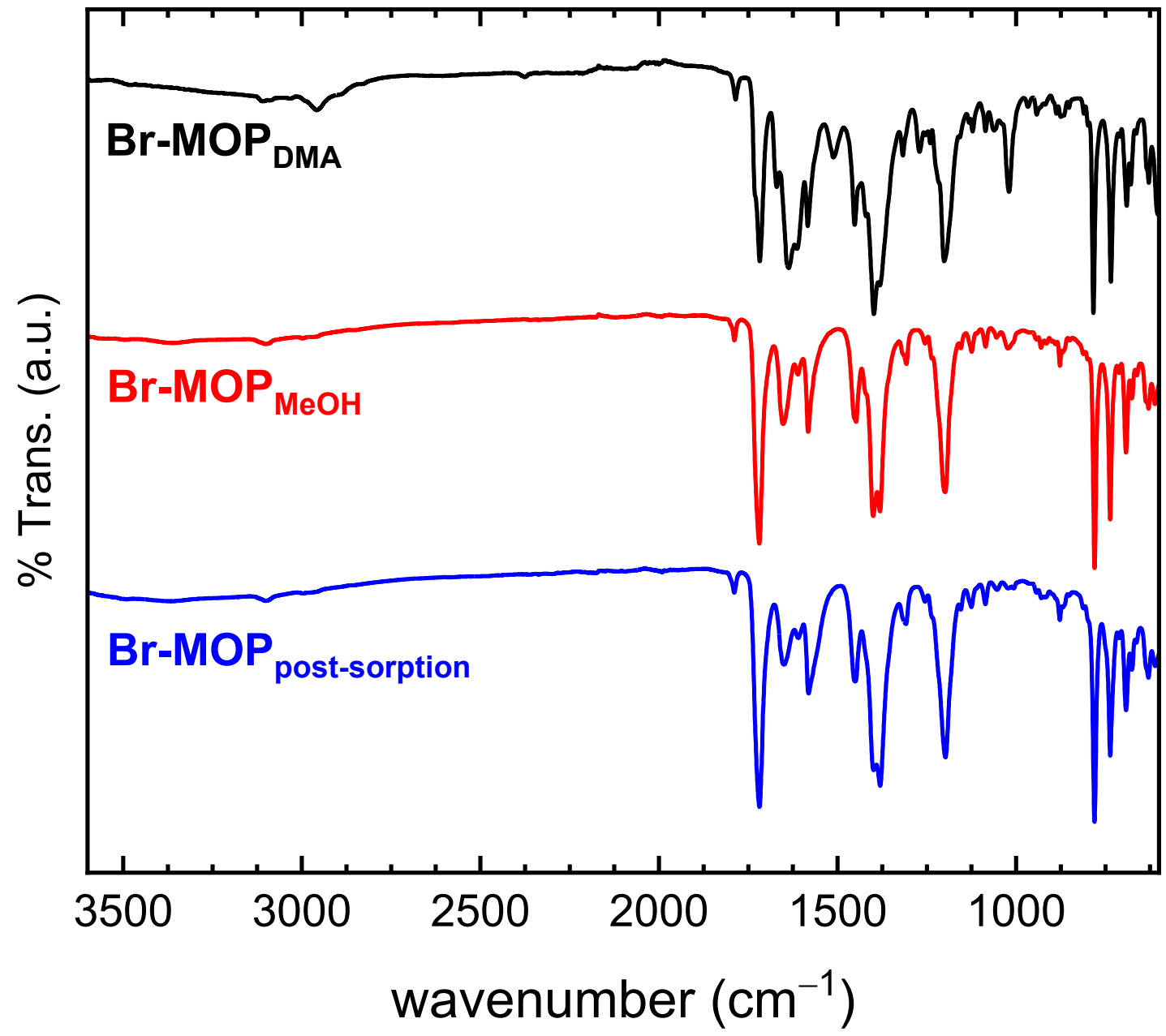

Figure S6. IR spectra of various phases of **Br-MOP**. (top) The as-synthesised phase **Br-MOP$_{DMA}$**; (middle) **Br-MOP$_{MeOH}$**; (bottom) **Br-MOP** after gas sorption measurements.

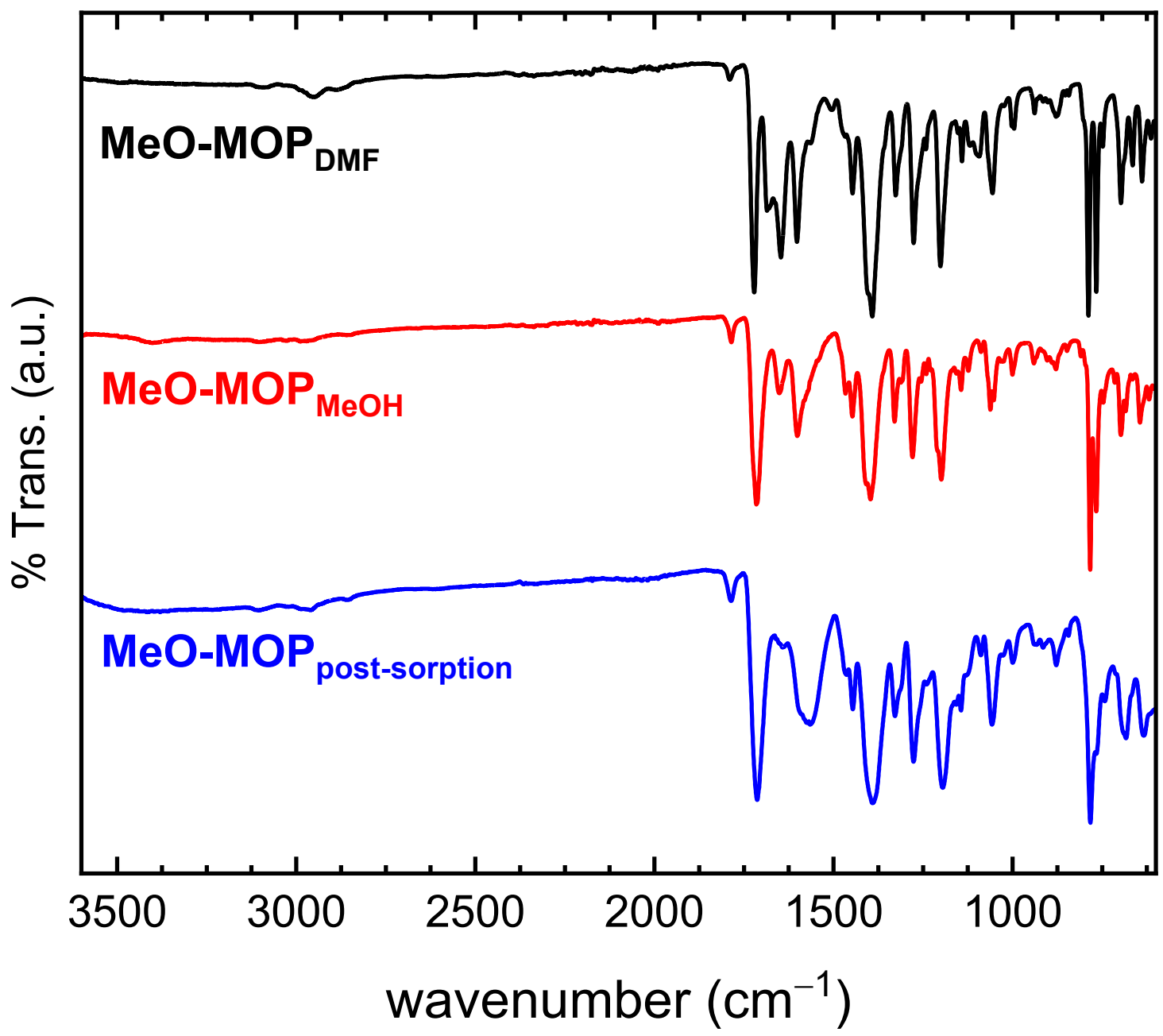

Figure S7. IR spectra of various phases of **MeO-MOP**. (top) The as-synthesised phase **MeO-MOP$_{DMF}$**; (middle) Solvent exchanged **MeO-MOP$_{MeOH}$**; (bottom) post gas sorption measurements.

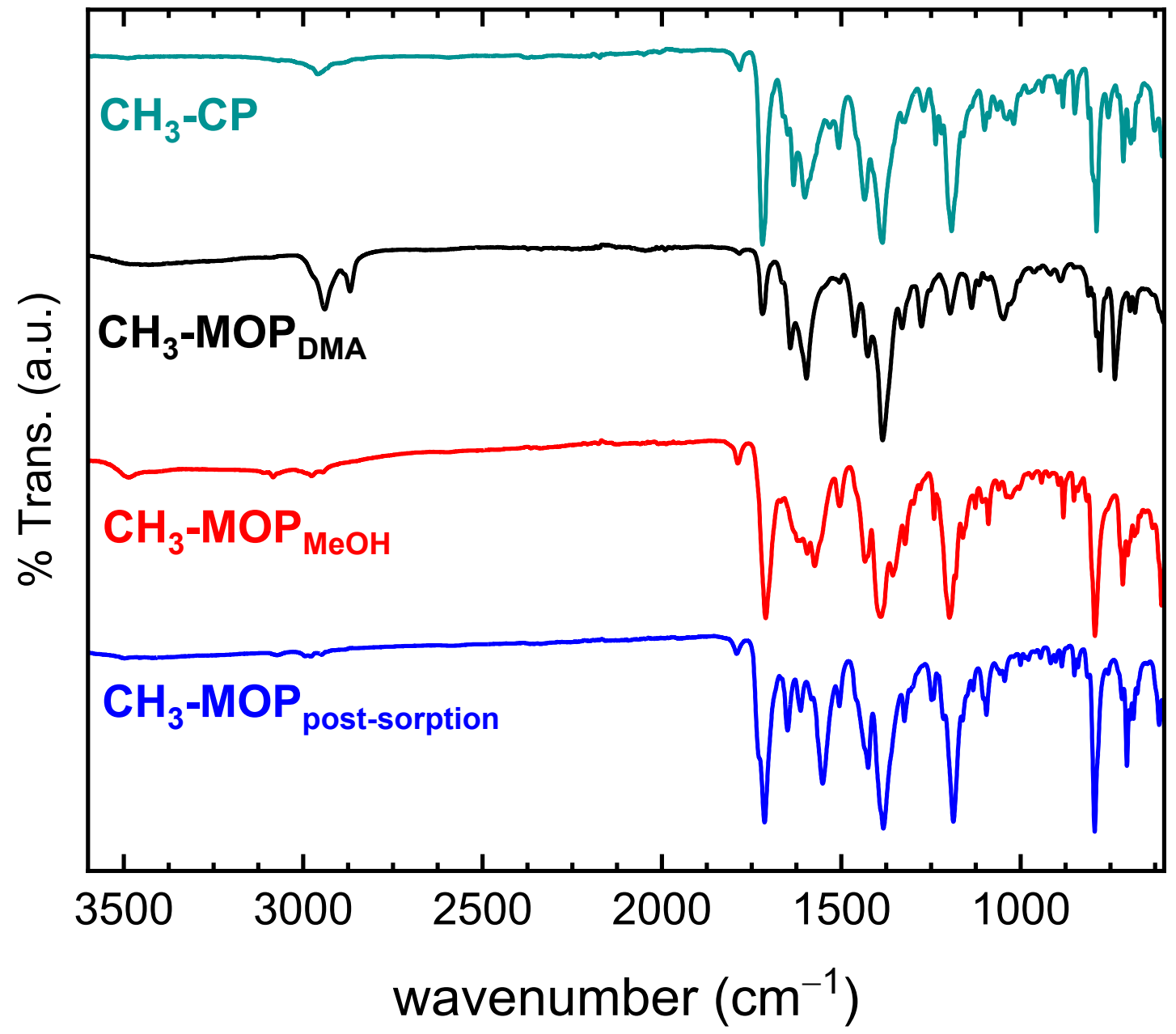

Figure S8. IR spectra of various phases of **CH$_3$-MOP** and the IR spectrum of **CH$_3$-CP**. (top) The as-synthesised phase **CH$_3$-CP**; (second top) the as-synthesised phase of **CH$_3$-MOP$_{DMA}$**; (second bottom) **CH$_3$-MOP$_{MeOH}$**; (bottom) **CH$_3$-MOP** after gas sorption measurements.

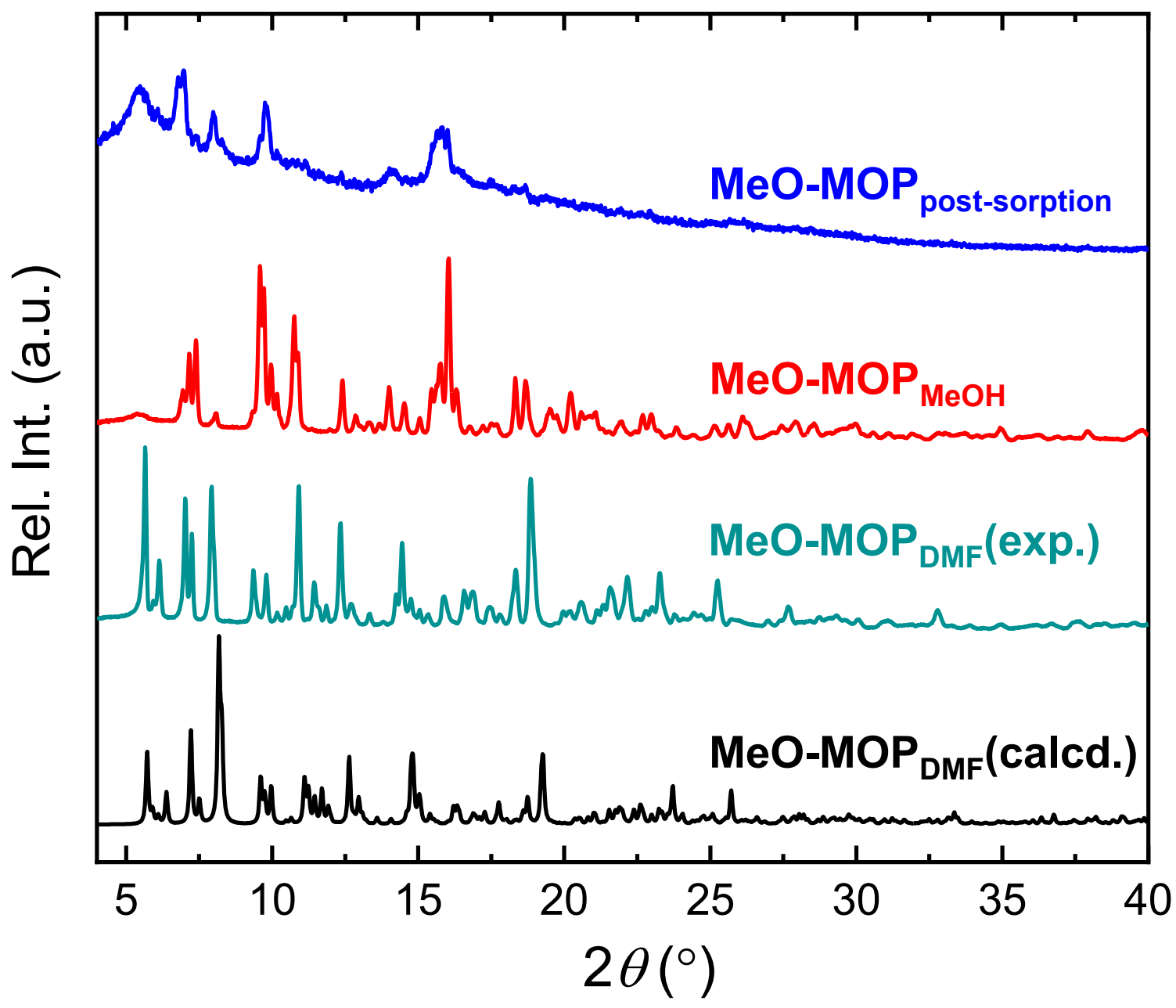

Figure S9. PXRD data for the various phases of **MeO-MOP**. (Bottom) The simulated pattern from the SXRD structure; (second bottom) the experimental data for the as-synthesised crystals; (second top) the experimental data for the solvent exchanged phase; and (top) the PXRD data collected after gas sorption measurements.

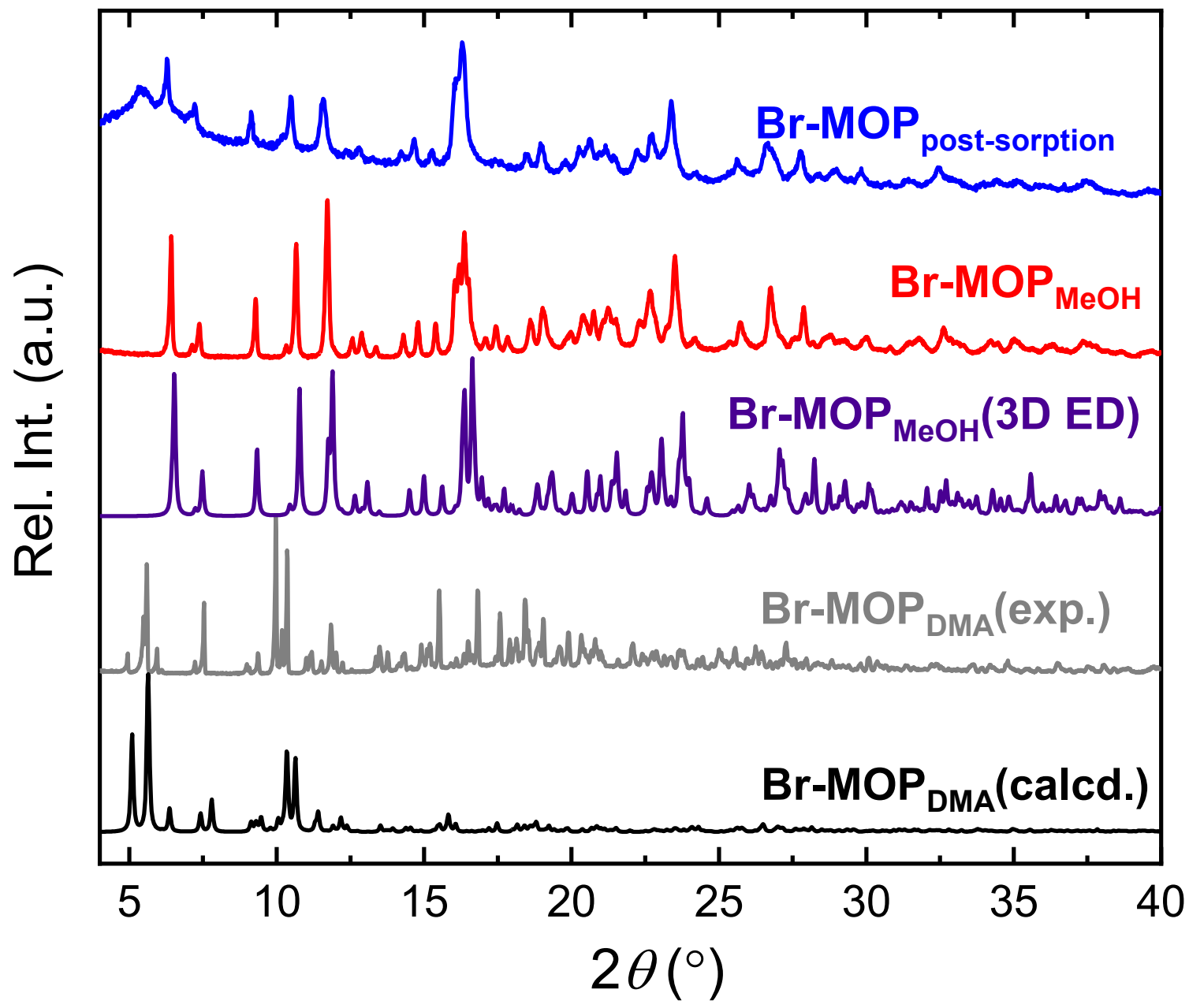

Fig. S10. PXRD data for the various phases of **Br-MOP**. (Bottom) The simulated pattern from the SXRD structure; (second bottom) the experimental data for the as-synthesised crystals; (middle) the simulated pattern based on the 3D ED structure of the phase exchanged with MeOH; (second top) experimental data for the solvent exchanged phase; and (top) the PXRD data collected after gas sorption measurements.

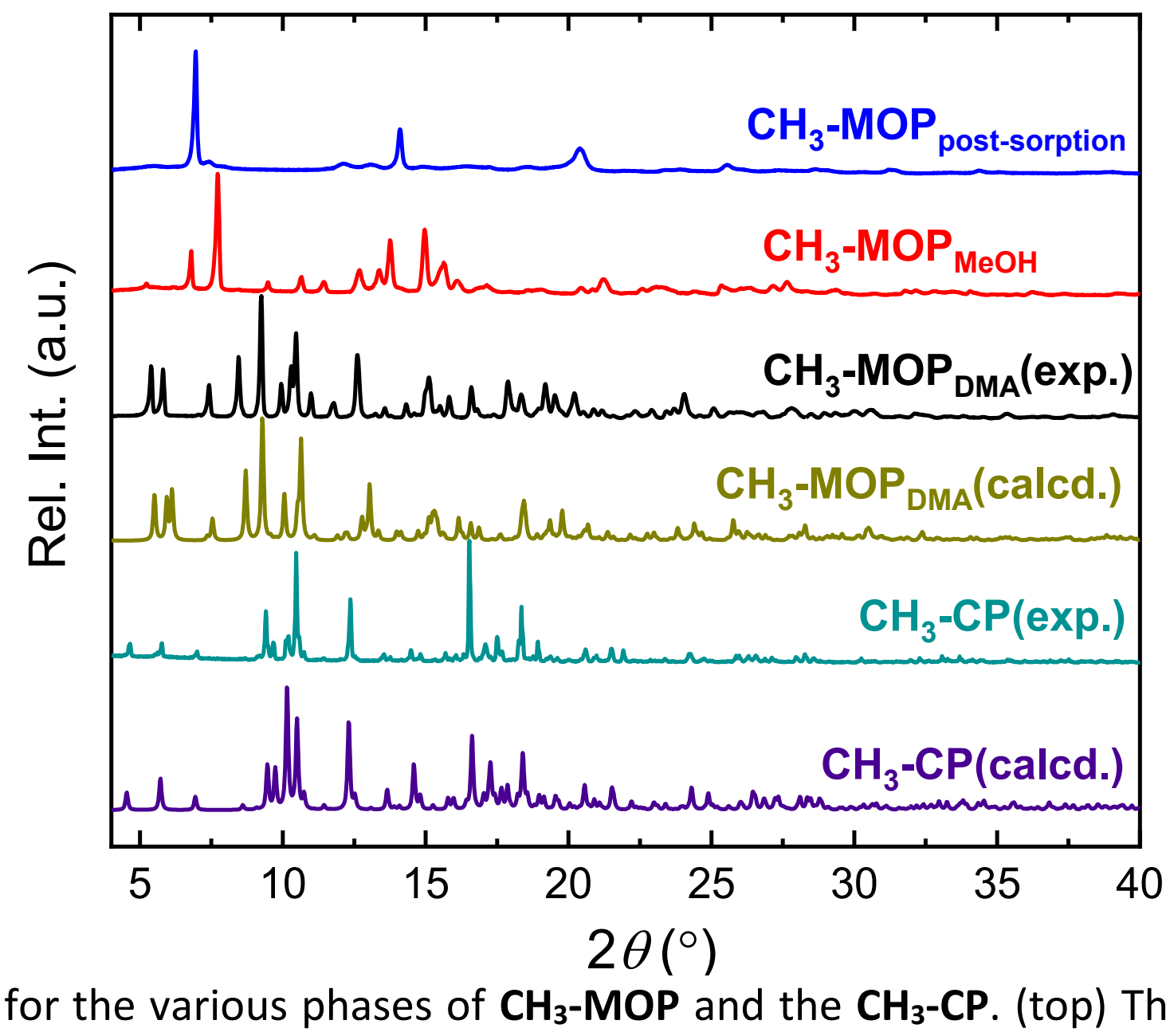

Figure S11. PXRD data for the various phases of **CH_3-MOP** and the **CH_3-CP**. (top) The as-synthesised phase **CH_3-CP**; (second top) the as-synthesised phase of **CH_3-MOP$_{DMA}$**; (second bottom) **CH_3-MOP$_{MeOH}$**; (bottom) **CH_3-MOP** after gas sorption measurements.

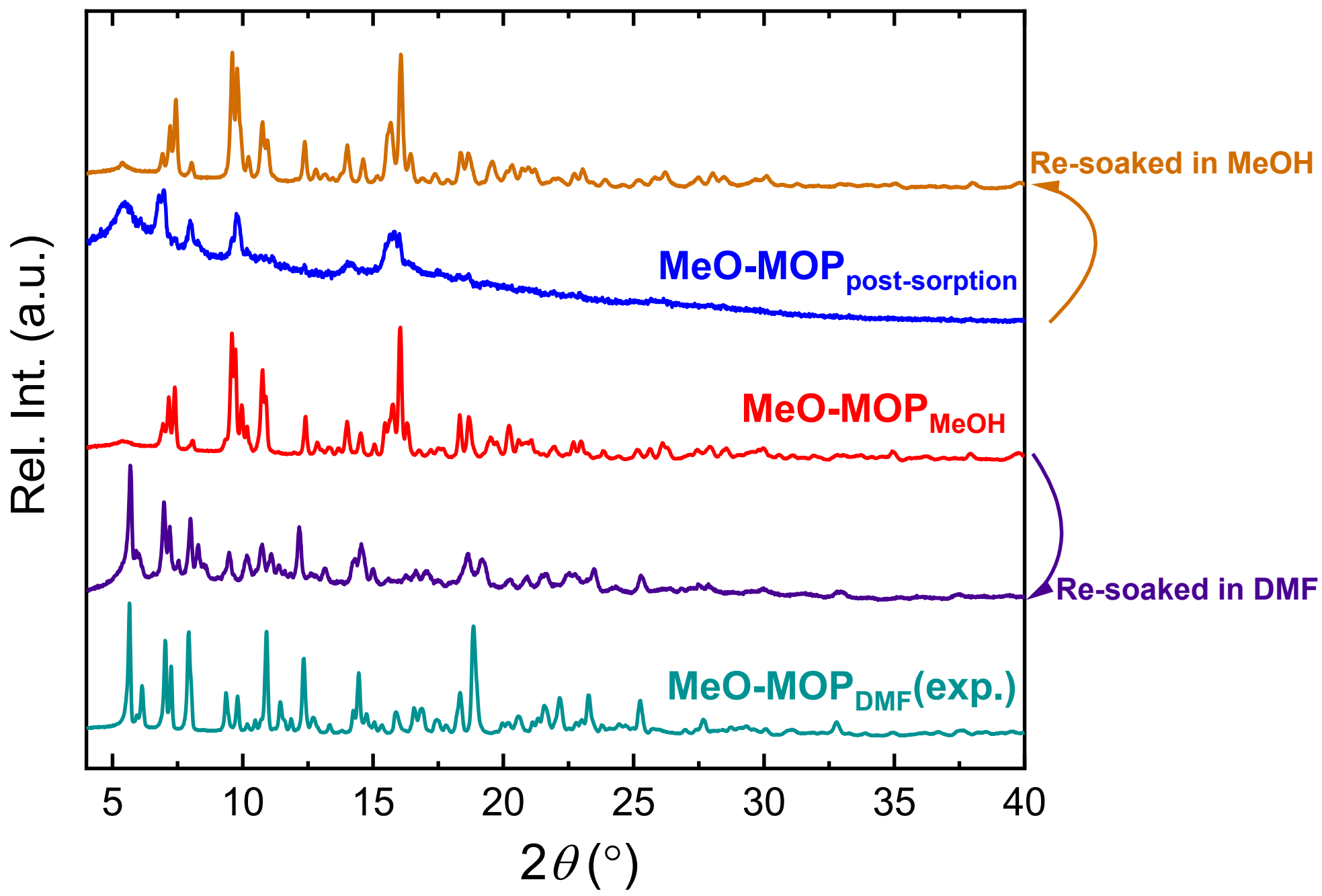

Fig. S12. PXRD data for the reversibility of the phase transitions seen in **MeO-MOP**. (Bottom) The crystallinity of the post-sorption phase is fully recovered upon soaking in MeOH. **MeO-MOP$_{DMF}$** can be recovered by soaking the MeOH solvate in DMF, albeit the phase recovered has lower crystallinity.

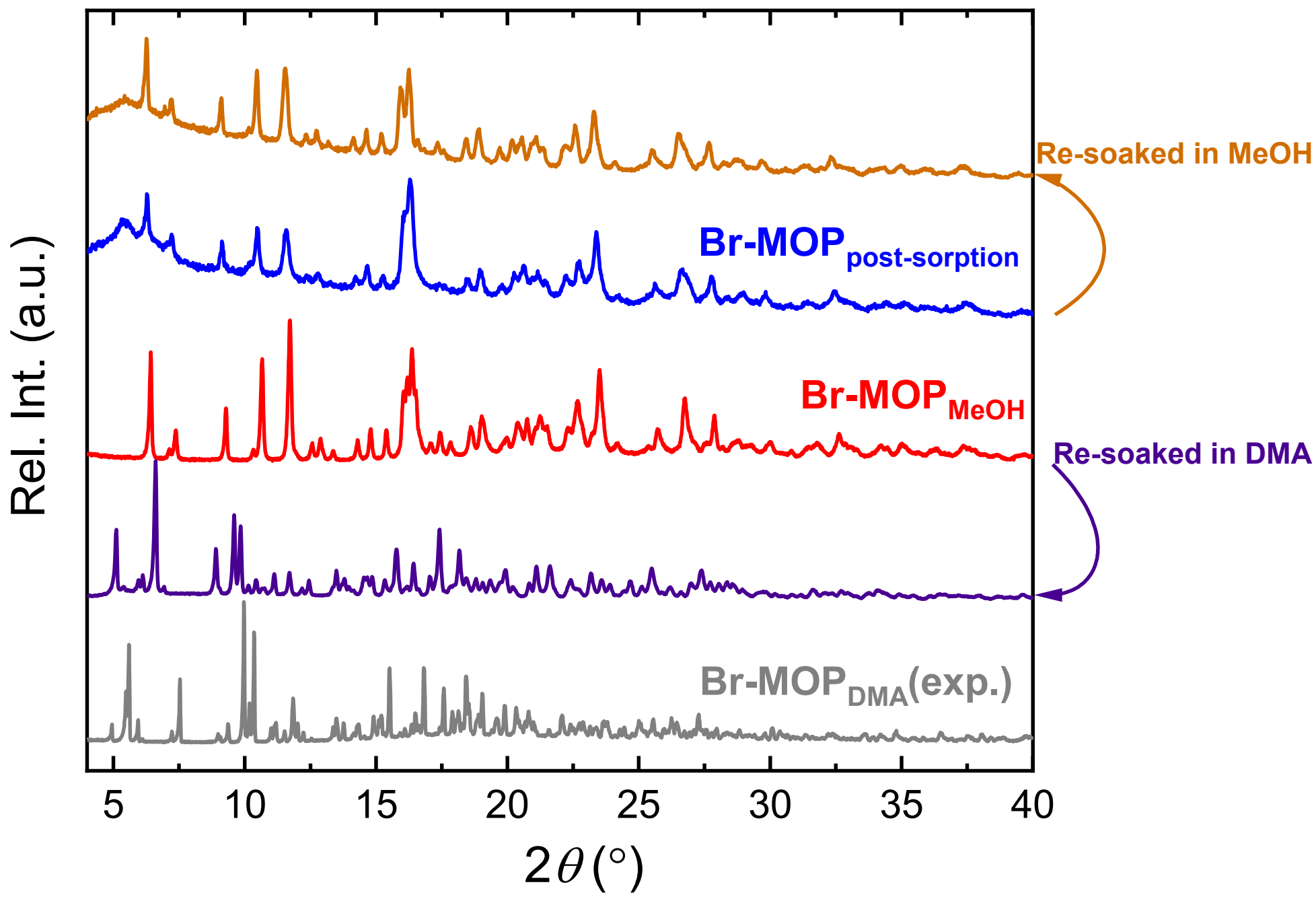

Fig. S13. PXRD data for the reversibility of the phase transitions seen in **Br-MOP**. (Bottom) The crystallinity of the post-sorption phase can be improved with repeated soaking in MeOH (here shown after two exchanges). **Br-MOP$_{MeOH}$** can be soaked in DMA, to induce a partial recovery of the initial solvated phase. Some of the cage remains in DMA due to its solubility, meaning the recovered solid presents a mixtures of phases.

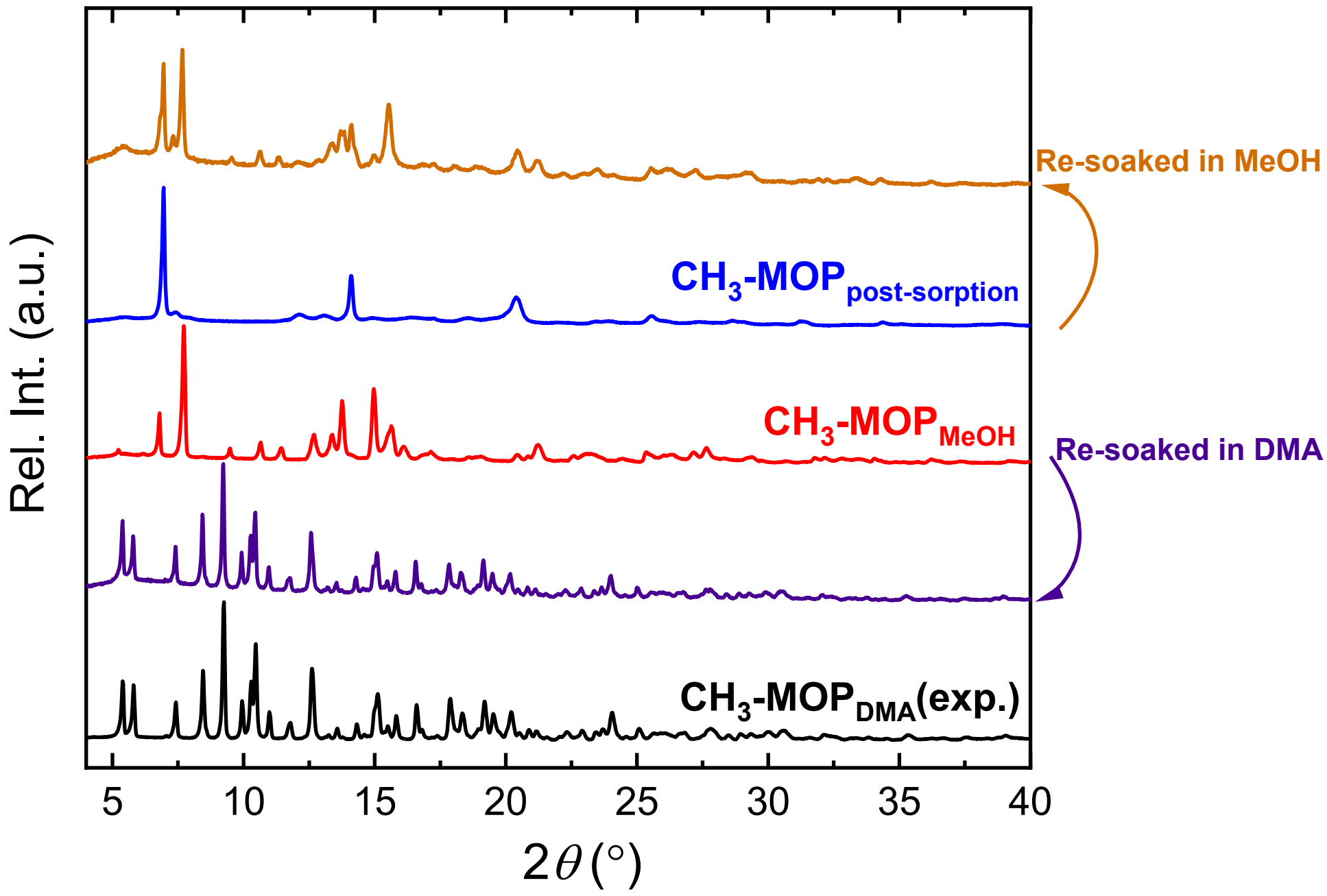

Fig. S14. PXRD data for the reversibility of the phase transitions seen in **CH_3-MOP**. (Bottom) The initial MeOH solvate phase can be partially recovered up on re-soaking the post-sorption sample in MeOH. **CH_3-MOP$_{DMA}$** can be fully recovered by soaking the MeOH solvate in DMA.

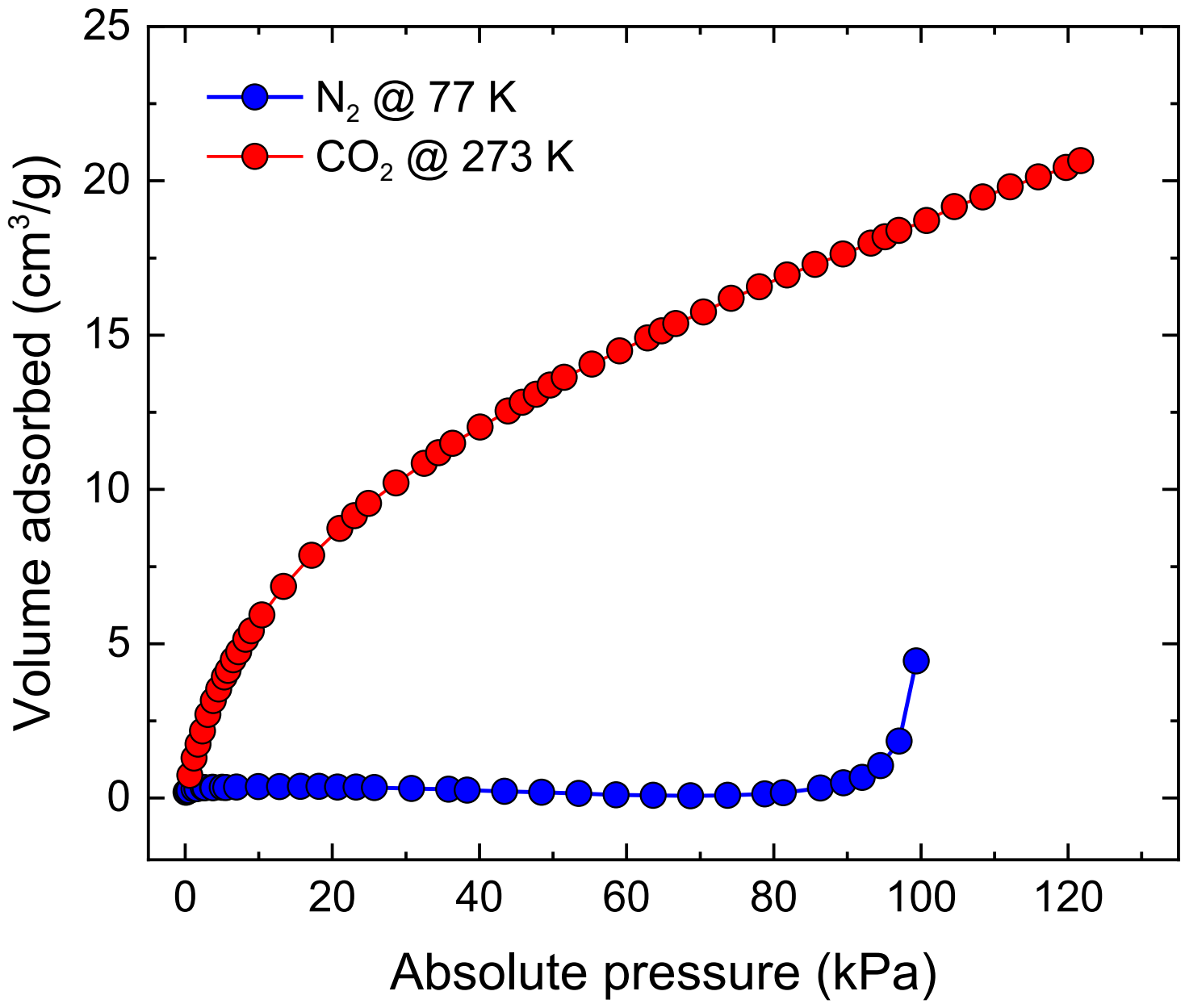

Figure S15. Adsorption isotherms for **MeO-MOP**, after activation under vacuum at 393 K.

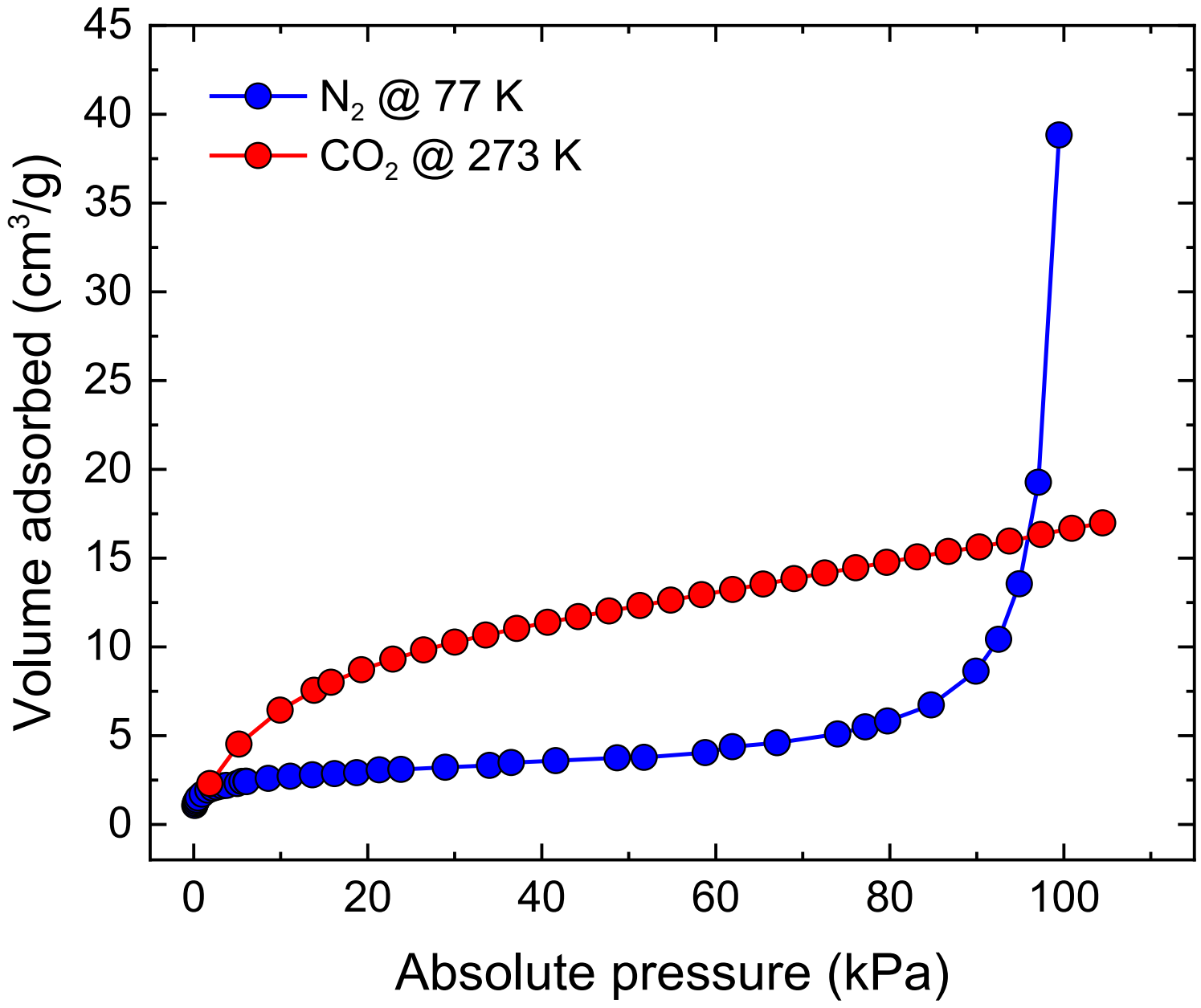

Figure S16. Adsorption isotherms for **Br-MOP**, after activation under vacuum at 393 K.

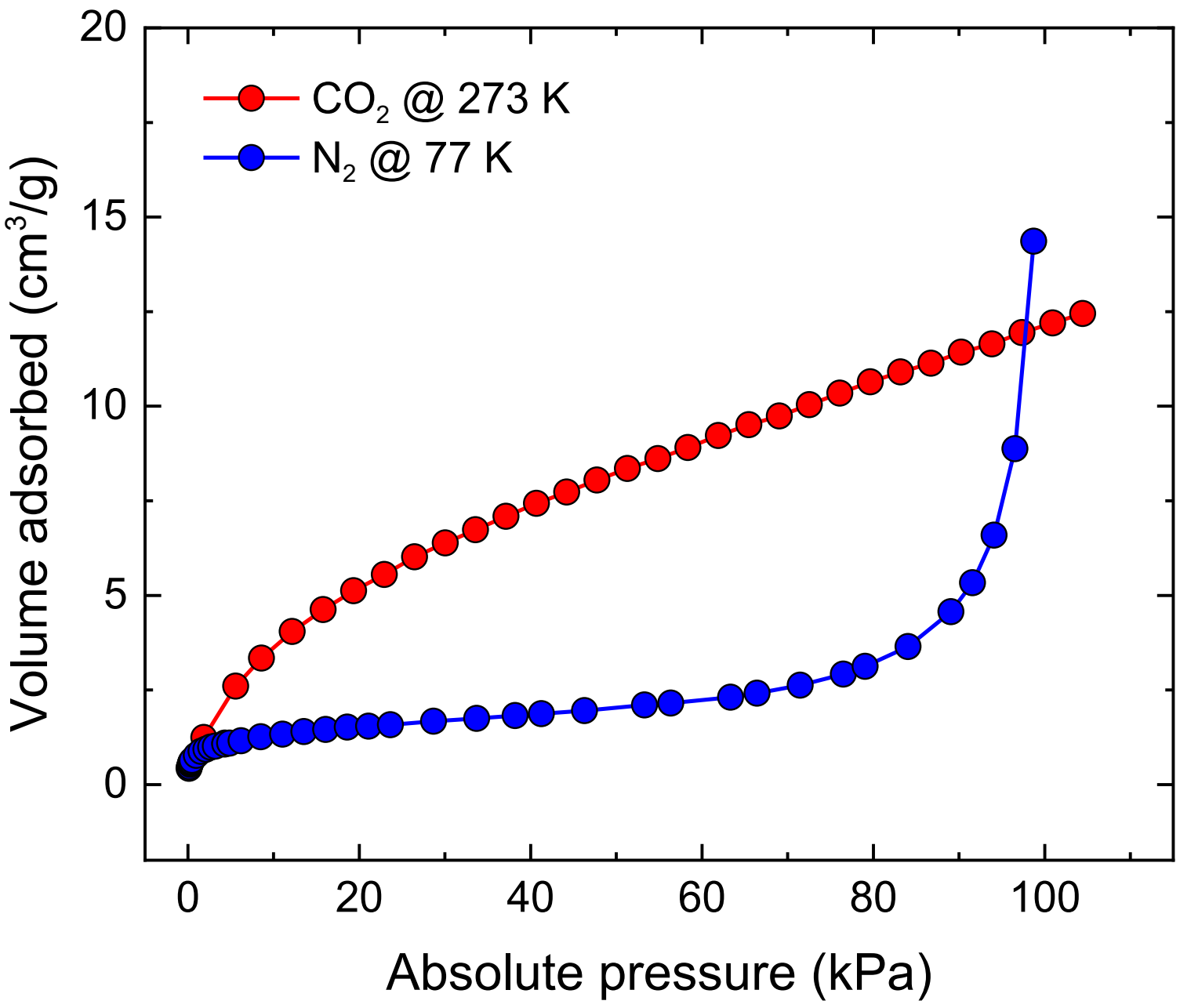

Figure S17. Adsorption isotherms for **CH_3-MOP**, after activation under vacuum at 393 K.

References

1 B. Doñagueda Suso, A. Legrand, C. Weetman, A. R. Kennedy, A. J. Fletcher, S. Furukawa and G. A. Craig, *Chem. Eur. J.*, 2023, **29**, e202300732.
2 CrysAlisPro Rigaku Oxford Diffraction 2025.
3 G. M. Sheldrick, *Acta Cryst.*, 2015, **C71**, 3–8.
4 O. V. Dolomanov, L. J. Bourhis, R. J. Gildea, J. A. K. Howard and H. Puschmann, *J. Appl. Cryst.*, 2009, **42**, 339–341.
5 L. Farrugia, *J. Appl. Cryst.*, 1999, **32**, 837–838.
6 A. Spek, *Acta Cryst.*, 2015, **C71**, 9–18.
7 L. J. Bourhis, O. V. Dolomanov, R. J. Gildea, J. A. K. Howard and H. Puschmann, *Acta Cryst.*, 2015, **A71**, 59–75.

8 A. Saha, S. S. Nia and J. A. Rodríguez, *Chem. Rev.*, 2022, **122**, 13883–13914.
9 A. Thorn, B. Dittrich and G. M. Sheldrick, *Acta Cryst.*, 2012, **A68**, 448–451.
